# Deep learning analysis of blood flow sounds to detect arteriovenous fistula stenosis

**DOI:** 10.1038/s41746-023-00894-9

**Published:** 2023-09-01

**Authors:** George Zhou, Yunchan Chen, Candace Chien, Leslie Revatta, Jannatul Ferdous, Michelle Chen, Shourov Deb, Sol De Leon Cruz, Alan Wang, Benjamin Lee, Mert R. Sabuncu, William Browne, Herrick Wun, Bobak Mosadegh

**Affiliations:** 1 Weill Cornell Medicine, New York, NY 10021 USA; 2 City University of New York, Hunter College, New York, NY 10021 USA; 3 School of Electrical and Computer Engineering, Cornell University and Cornell Tech, New York, NY 10044 USA; 4 Department of Radiology, Weill Cornell Medicine, New York, NY 10021 USA; 5 Department of Interventional Radiology, NewYork-Presbyterian Hospital, New York, NY 10021 USA; 6 Department of Vascular Surgery, NewYork-Presbyterian Hospital, New York, NY 10021 USA; 7 Dalio Institute of Cardiovascular Imaging, Department of Radiology, Weill Cornell Medicine, New York, NY 10021 USA

**Keywords:** Machine learning, Kidney diseases

## Abstract

For hemodialysis patients, arteriovenous fistula (AVF) patency determines whether adequate hemofiltration can be achieved, and directly influences clinical outcomes. Here, we report the development and performance of a deep learning model for automated AVF stenosis screening based on the sound of AVF blood flow using supervised learning with data validated by ultrasound. We demonstrate the importance of contextualizing the sound with location metadata as the characteristics of the blood flow sound varies significantly along the AVF. We found the best model to be a vision transformer trained on spectrogram images. Our model can screen for stenosis at a performance level comparable to that of a nephrologist performing a physical exam, but with the advantage of being automated and scalable. In a high-volume, resource-limited clinical setting, automated AVF stenosis screening can help ensure patient safety via early detection of at-risk vascular access, streamline the dialysis workflow, and serve as a patient-facing tool to allow for at-home, self-screening.

## Introduction

The arteriovenous fistula (AVF) is often touted as the “lifeline” for dialysis patients. According to the National Kidney Foundation (NKF), vascular access is globally ranked as a top priority for dialysis patients, healthcare providers, and clinical research^1^. Preserving dialysis access is a high priority for providers and patients because the consequences of AVF dysfunction and subsequent access failure significantly contributes to patient morbidity and healthcare costs. Unfortunately, AVF dysfunction is not uncommon. One 5-year study from 2018 that analyzed AVF failures found a cumulative patency loss rate of 19.7% and 33.3% during the early and late period, respectively^2^. According to the United States Renal Data System (USRDS), from 2016–2018, the cumulative incidence of loss of primary unassisted patency at 1 year was 51.8%, the loss of primary assisted patency was 19.0%, and the loss of secondary patency was 3.3%. It is well documented that the most common cause of AVF dysfunction and subsequent failure is stenosis and thrombosis^3–6^. One study found that the incidence of stenosis is 4.6–10.8%, and the incidence of thrombosis is 2.3–7.7%^7^. Nearly all thrombosed AVFs have an underlying stenotic lesion^8^. While patients on hemodialysis are in a general prothrombotic state, which increases the risk for stroke and ischemic heart disease, studies have found that vascular access-related complications are the leading cause of hospitalizations among dialysis patients^9^. Once there is access site thrombosis, urgent intervention is required for salvage in order to prevent permanent loss of the AVF.

Vascular access complications, such as stenosis and thrombosis, are significant drivers of resource utilization, cost, morbidity, and mortality^10–14^. Screening for AVF stenosis improves the longevity of AVFs, reduce costs for healthcare systems, and improve the quality of life for patients. The current Kidney Disease Outcomes Quality Initiative (KDOQI) guidelines recommend screening for AVF stenosis through “the examination and evaluation of the access by means of physical examination to detect clinical signs that suggest the presence of AV access flow dysfunction”^15^. A lesion is considered clinically significant if it contributes to clinical signs and symptoms, such as arm swelling, prolonged bleeding after dialysis, or changes in the access bruit (rumbling sound) or thrill (tactile sensation); regardless of sustained changes in measurements such as access flow or venous pressures^16–18^.

Auscultation (i.e., listening for internal body sounds) is a noninvasive method, compared to digital subtraction angiography or venous cannulation, and more convenient compared to ultrasound for detecting abnormal blood flow^19^. Additionally, a change in access bruit or thrill may be one of the earliest clinical indicators that a stenosis is developing and can be measured using a low-cost and widely available digital stethoscope. However, the reality is that auscultation is a highly subjective physical exam technique and largely depends on the skill of the listener^20–23^. Since the timely diagnosis of stenosis is crucial for maintaining dialysis access, applying deep learning to AVF blood flow sounds can enhance the ability of healthcare providers to screen for AVF stenosis both reliably and efficiently.

**Fig. 1:**
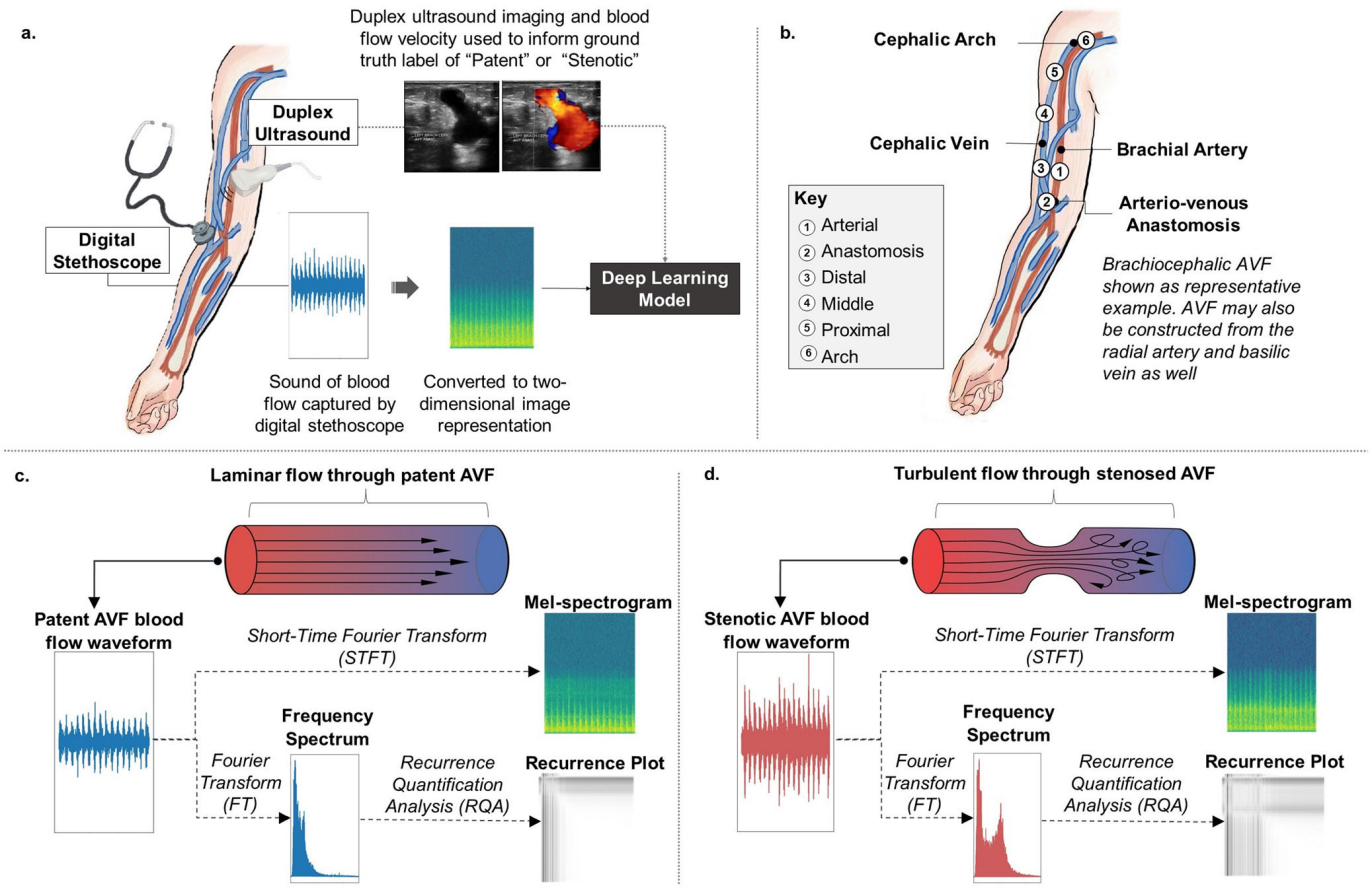
Schematic of overall project. **a** Sound of blood flow captured by digital stethoscope. The one-dimensional blow flow audio signal is preprocessed into two-dimensional image representations, which were used to train the deep learning models investigated in this paper. Ultrasound imaging and blood flow velocities measured by concurrent duplex ultrasound were used to inform the binary ground truth label of either “Patent” or “Stenotic”. The deep learning models are trained following the supervised learning paradigm. **b** The 6 locations along the arteriovenous fistula from where blood flow sounds are collected numbered in increasing order from most distal to most proximal based on the anatomic definitions of the arm: artery, anastomosis (where the artery joins the vein), the distal vein, the middle vein, the proximal vein, and the arch of the vein. Shown in this illustration is the brachiocephalic fistula, but the brachiobasilic, radiocephalic, and radiobasilic fistula is also studied in this paper. **c**, **d** Laminar flow through a patent arteriovenous fistula (AVF) generates a quiet “whooshing” sound. As an AVF develops stenosis, laminar flow will transition to turbulent flow. Increasing turbulent flow will result in an increased amount of higher frequency components in the generated sound. Clinically, the sound heard when auscultating a stenosed AVF is often described as a “high-pitched systolic bruit or thrill”. The two image representations of sound explored in this study are the mel-spectrogram and the recurrence plot. The mel-spectrogram is generated from applying the short-time Fourier Transform (STFT) to the waveform. The recurrence plot is generated from a recurrence quantification analysis (RQA) of the frequency spectrum, which is obtained from applying the Fourier Transform (FT) on the waveform. The illustrative example patent and stenotic waveforms, frequency spectrums, mel-spectrograms, and recurrence plots seen here are taken from a patent and stenotic “proximal” vein, respectively.

In this Article, blood flow sounds are recorded using a digital stethoscope at six distinct locations along each patient’s AVF. The overall schematic of our project is demonstrated in Fig. 1. We choose to pre-process the recorded one-dimensional blood flow audio signals into two-dimensional image representations to leverage the state-of-the-art models developed by the computer vision community. We trained our models using supervised learning with labels validated from concurrent duplex ultrasound. We found that these models could better predict patients with a stenosis compared to non-machine learning analyses of the same sound files. A deep learning model trained on normal and abnormal blood flow sounds that can identify AVF stenosis could establish a level of objectivity to the subjective interpretation of auscultated sounds via the extraction and quantification of relevant features from the blood flow audio signals. Deep learning has already been successfully utilized to help predict AVF failure and successful maturation based on various patient parameters^24,25^. Additionally, deep learning affords a level of automation over the screening process. Our proposed technology could even serve as a patient-facing tool to allow for at-home, self-screening of AVF stenosis. This ability could be especially helpful in under-resourced areas where patients may not be receiving routine screening. The timely and accurate detection of AVF stenosis using deep learning analysis of AVF blood flow sounds can reduce downstream healthcare costs, and more importantly, improve the quality of life of patients.

## Results

### Data

Table 1 summarizes the demographic and clinical characteristics of the patients enrolled in our study.

**Table 1:** Clinical and demographic characteristics of the patients included in this study.

	Patent (*N* = 2113)	Stenotic (*N* = 452)	Overall (*N* = 2565)
Demographics			
Sex			
Male	1316 (62.3%)	285 (63.1%)	1601 (62.4%)
Female	797 (37.7%)	167 (36.9%)	964 (37.6%)
BMI			
Mean (SD)	25.0 (5.03)	24.6 (3.91)	24.9 (4.85)
Median [min, max]	24.2 [16.1, 66.7]	24.1 [16.1, 40.1]	24.2 [16.1, 66.7]
Age at AVF creation			
Mean (SD)	66.0 (13.9)	66.3 (13.9)	66.1 (13.9)
Median [min, max]	67.0 [18.0, 91.0]	67.0 [18.0, 91.0]	67.0 [18.0, 91.0]
AVF characteristics			
AVF velocity			
Mean (SD)	224 (125)	611 (130)	292 (194)
Median [min, max]	194 [14.0, 639]	620 [0, 984]	233 [0, 984]
AVF type			
Brachiocephalic	1202 (56.9%)	250 (55.3%)	1452 (56.6%)
Radiocephalic	405 (19.2%)	99 (21.9%)	504 (19.6%)
Brachiobasilic	408 (19.3%)	79 (17.4%)	487 (19.0%)
Radiobasilic	98 (4.6%)	24 (5.3%)	122 (4.8%)
Past medical history			
Comorbidities			
Hypertension	2030 (96.1%)	437 (96.7%)	2467 (96.2%)
Cardiovascular disease	1565 (74.1%)	338 (74.8%)	1903 (74.2%)
Peripheral artery disease	1229 (58.2%)	271 (60.0%)	1500 (58.5%)
Diabetes	1118 (52.9%)	222 (49.1%)	1340 (52.2%)
Deep vein thrombosis	8 (0.4%)	3 (0.7%)	11 (0.4%)
Smoking status			
Never	1414 (66.9%)	297 (65.7%)	1711 (66.7%)
Former	603 (28.5%)	127 (28.1%)	730 (28.5%)
Current	95 (4.5%)	28 (6.2%)	123 (4.8%)
Renal disease etiology			
ESRD, unspecified	1524 (72.1%)	339 (75.0%)	1863 (72.6%)
Hypertensive nephropathy	294 (13.9%)	46 (10.2%)	340 (13.3%)
Diabetic nephropathy	110 (5.2%)	19 (4.2%)	129 (5.0%)
Polycystic kidney disease	71 (3.4%)	22 (4.9%)	93 (3.6%)
Chronic glomerulonephritis	43 (2.0%)	9 (2.0%)	52 (2.0%)
IgA nephropathy	35 (1.7%)	10 (2.2%)	45 (1.8%)
Renal cancer	23 (1.1%)	7 (1.5%)	30 (1.2%)
Congenital etiologies	12 (0.6%)	0 (0%)	12 (0.5%)
Complications			
AVF revision			
No	551 (26.1%)	132 (29.2%)	683 (26.6%)
Yes	1556 (73.6%)	319 (70.6%)	1875 (73.1%)
Number of revisions			
Mean (SD)	3.45 (4.77)	3.27 (4.82)	3.42 (4.78)
Median [min, max]	2.00 [0, 31.0]	2.00 [0, 31.0]	2.00 [0, 31.0]
Month to earliest revision			
Mean (SD)	14.5 (24.0)	14.7 (27.0)	14.5 (24.5)
Median [min, max]	5.00 [1.00, 143]	4.00 [1.00, 143]	5.00 [1.00, 143]
Physical exam			
Heart rate at visit			
Mean (SD)	77.9 (11.9)	78.1 (11.9)	77.9 (11.9)
Median [min, max]	76.0 [52.0, 118]	76.0 [52.0, 118]	76.0 [52.0, 118]
Systolic blood pressure at visit			
Mean (SD)	136 (20.1)	139 (20.5)	137 (20.2)
Median [min, max]	138 [90.0, 182]	140 [90.0, 182]	138 [90.0, 182]
Diastolic blood pressure at visit			
Mean (SD)	72.1 (11.1)	73.6 (11.7)	72.4 (11.3)
Median [min, max]	71.0 [43.0, 102]	72.0 [43.0, 102]	71.0 [43.0, 102]

Table 2 gives a breakdown of the distribution of stenotic and patent AVFs by location.

**Table 2:** Breakdown of patent versus stenotic lesions per location (based on the anatomic definitions of the arm).

AVF Location	Status	Count
Artery		
	Patent	441 (99%)
	Stenotic	6 (1%)
Anastomosis		
	Patent	424 (88%)
	Stenotic	57 (12%)
Distal vein		
	Patent	252 (54%)
	Stenotic	213 (46%)
Middle vein		
	Patent	399 (89%)
	Stenotic	48 (11%)
Proximal vein		
	Patent	332 (84%)
	Stenotic	62 (16%)
Venous arch		
	Patent	240 (81%)
	Stenotic	55 (19%)
Total		
	Patent	2088 (83%)
	Stenotic	441 (17%)

### Frequency spectrums

**Fig. 2:**
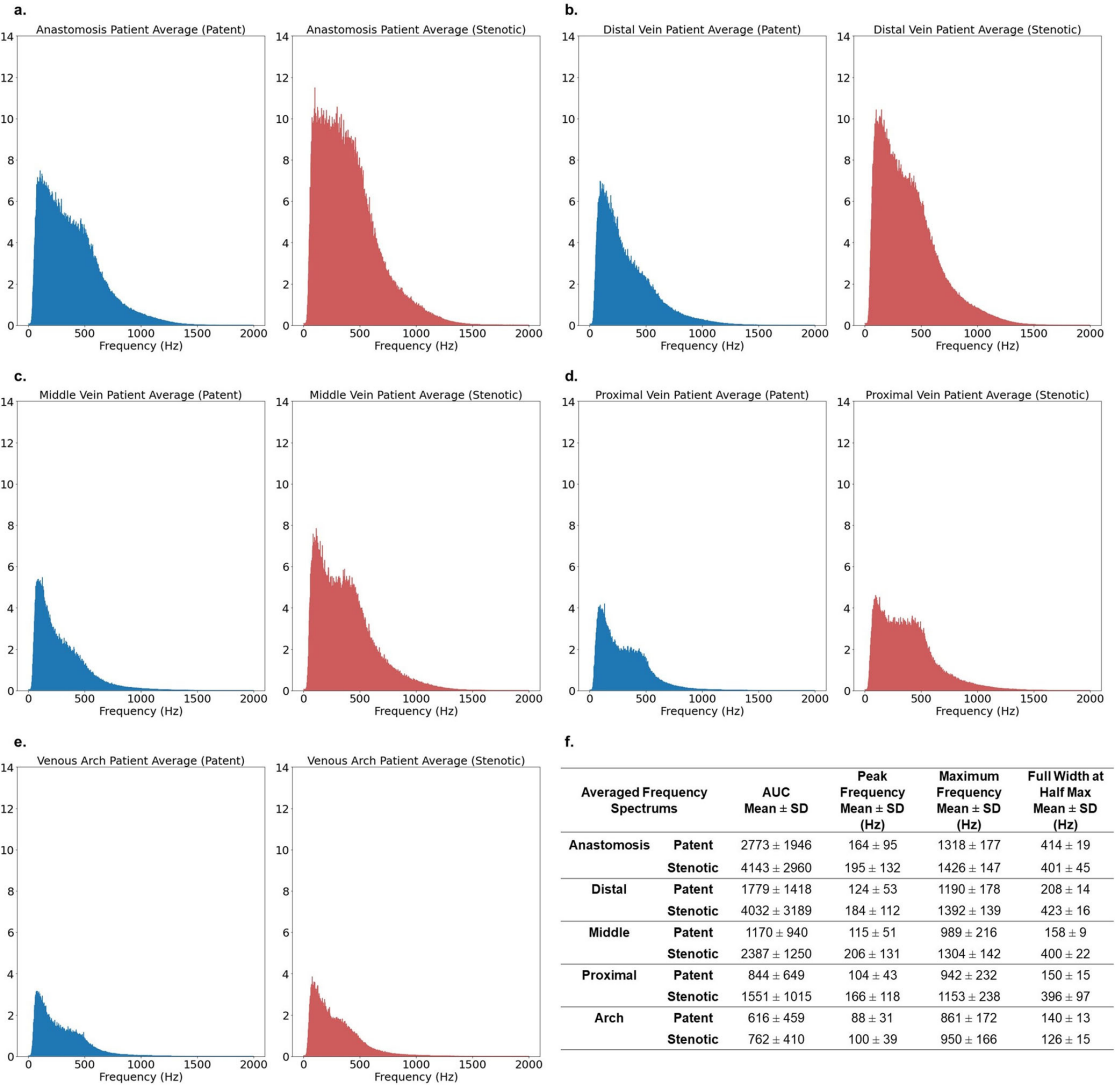
Averaged patent and stenotic frequency spectrums across all patients, stratified by location. We computed the averaged frequency spectrum of blood flow sounds for patent (blue) and stenotic (red) fistulas across all patients in the training and validation sets (311 patients total) at **a** the anastomosis site, **b** the distal vein site, **c** the middle vein site, **d** the proximal vein site, **e** and the venous arch site. **f** Descriptive, numerical summary of the averaged frequency spectrums include the area under the curve (AUC), peak frequency, maximum frequency, and full width at half max.

To gain some intuition about how the blood flow sounds differs by location along the AVF and how patent and stenotic sounds differ from each other at each location, we computed the averaged frequency spectrum across all patients in the training set. We also derived scalar metrics from the averaged frequency spectrums including the area under the curve, peak frequency, maximum frequency, and full width at half max height. Fig. 2 displays the averaged frequency spectrums and quantitative scalar measures.

### Individual, location-based models

**Fig. 3:**
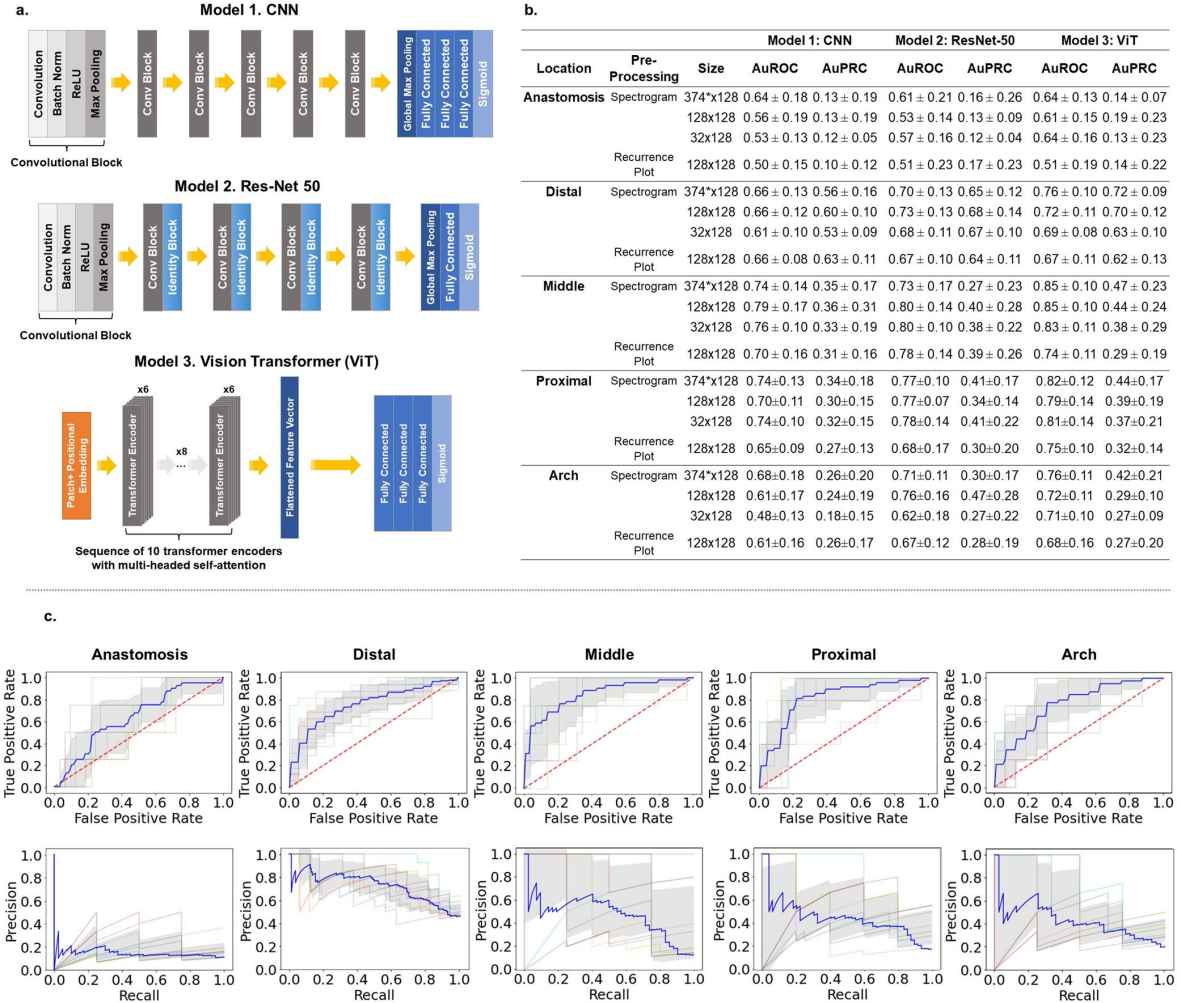
Schematic of model architectures and summary of results of location-based models. **a** The models explored in this study: a Convolutional Neural Network (CNN), a ResNet-50 pre-trained on ImageNet weights, a Vision Transformer (ViT). **b** Summary of results of Experiment 1: independent binary classifiers to distinguish patent vs stenotic at each location. In experiment 1, we compare the three model architectures and the two pre-processing methods – spectrograms and recurrence plot images – at each location. For the spectrogram images, we tested 3 different sizes of varying time resolution at the constant, maximum frequency resolution of 128: 374 × 128, 128 × 128, and 32 × 128. *Note that for the ViT, the 374 × 128 spectrogram image is resized to be 368 × 128 to be compatible with the 16 × 16 patch tokenization step. For the recurrence plot images, we used a resolution of 128 × 128. Model performance is quantified by the area under the receiver operating characteristics curve (AuROC) and the area under the precision recall curve (AuPRC) from 10-fold cross validation. **c** The ROC (top) and PR curves (bottom) for detecting stenosis at each location for the best performing model in Experiment 1: ViT trained on 368 × 128 spectrogram images. The ROC and PR curves for the other model architectures and pre-processing methods are shown in the Supplementary Figs. 3–8. The gray shading represents ± 1 standard deviation. Variance is calculated from the 10 different folds used in the 10-fold cross validation.

First, we studied binary classification of AVF blood flow sound at each location separately. We studied combinations of two different pre-processing methods with three different model architectures. The first method is to create a Mel-spectrogram image representation of the blood flow sound using a short-time Fourier transform. For the spectrogram image, we also explore three different time resolutions at the maximum frequency resolution. The second method is to create a recurrence plot image representation of the blood flow sound by applying recurrence quantification analysis to the signal in the frequency domain. Each image representation of sound is then used to train the three different model architectures. The first model is a 6-layer convolutional neural network (CNN). The second model is a ResNet-50 CNN pre-trained on ImageNet. The third model is a vision transformer (ViT). We refer to these models as “location-based models” since they are only trained on sounds from a single, given location. Fig. 3 depicts the model architectures and summary of the results for each pre-processing method and model architecture combination. For these individual, location-based models, we further study how important it is to contextualize these models with metadata regarding the anatomical origin of the artery and vein used to create the AVF. Results from these studies are depicted in Supplementary Figs. 12 & 13 and Supplementary Table 1.

### Universal model with and without location metadata

**Fig. 4:**
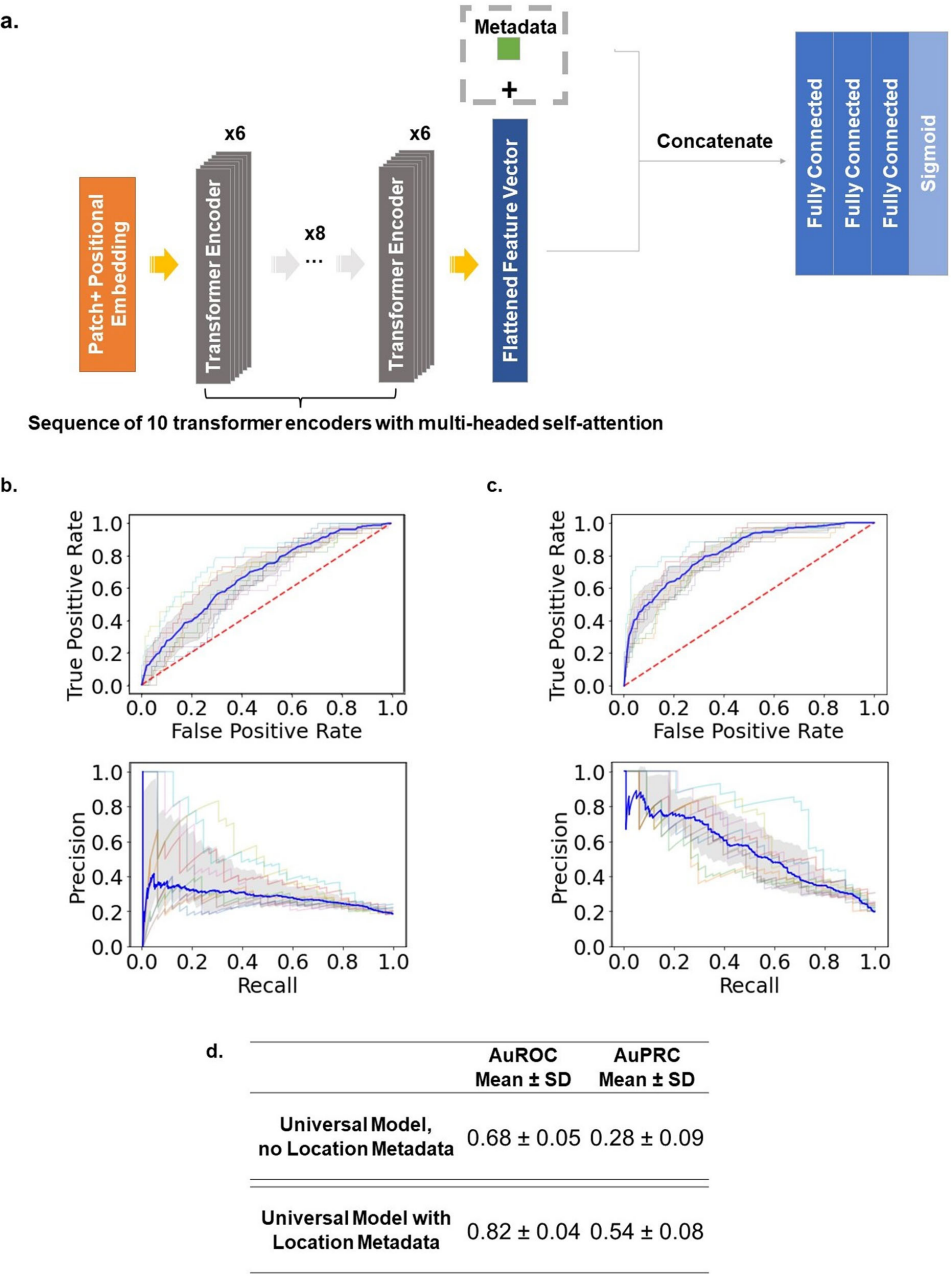
Universal Vision Transformer with and without location metadata. **a** Modified ViT architecture that also takes an encoded categorical input (i.e. location metadata) via concatenation to the flattened feature vector coming out of the last transformer encoder layer. **b** The ROC (top) and PR curves (bottom) for Experiment 2: universal binary classifier to distinguish patent vs stenotic, with no location metadata. The 368 × 128 spectrogram images from every location are aggregated together and used to train the conventional ViT (Model 3) without supplying the model any metadata about the location from which the spectrogram is sourced from. **c** The ROC (top) and PR curves (bottom) for Experiment 3 : universal binary classifier to distinguish patent vs stenotic, with location metadata. The 368 × 128 spectrogram images from every location are aggregated together to train the modified ViT (shown here), this time with location metadata supplied to the model. The categorical location information is first one-hot encoded, then fed into an embedding layer that converts the one-hot encoded vectors into a dense numerical vector representation that is then concatenated to the flattened feature vector. The embedding layer is trained along with the ViT. The gray shading represents ± 1 standard deviation. Variance is calculated from the 10 different folds used in the 10-fold cross validation. **d** Summary statistics of the universal model with and without location metadata. Results from other methods of encoding categorical information are shown in the Supplementary Fig. 11.

Next, we study the importance of contextualizing AVF blood flow sounds with location metadata. For this we study the ViT architecture trained on the Mel-spectrogram images. We refer to these models as “universal models” since they are trained on sounds from all the locations. In experiment II, we aggregate all the sounds from each location to train one ViT, but without any location metadata given to the model. In experiment III, we aggregate all the sounds from each location and supply location metadata to the ViT. We study various categorical encoding methods for encoding the location metadata including ordinal encoding, one-hot encoding, and learned embeddings. Fig. 4 shows the results from training our universal ViT with and without location metadata.

### Evaluation on held-out test set

**Fig. 5:**
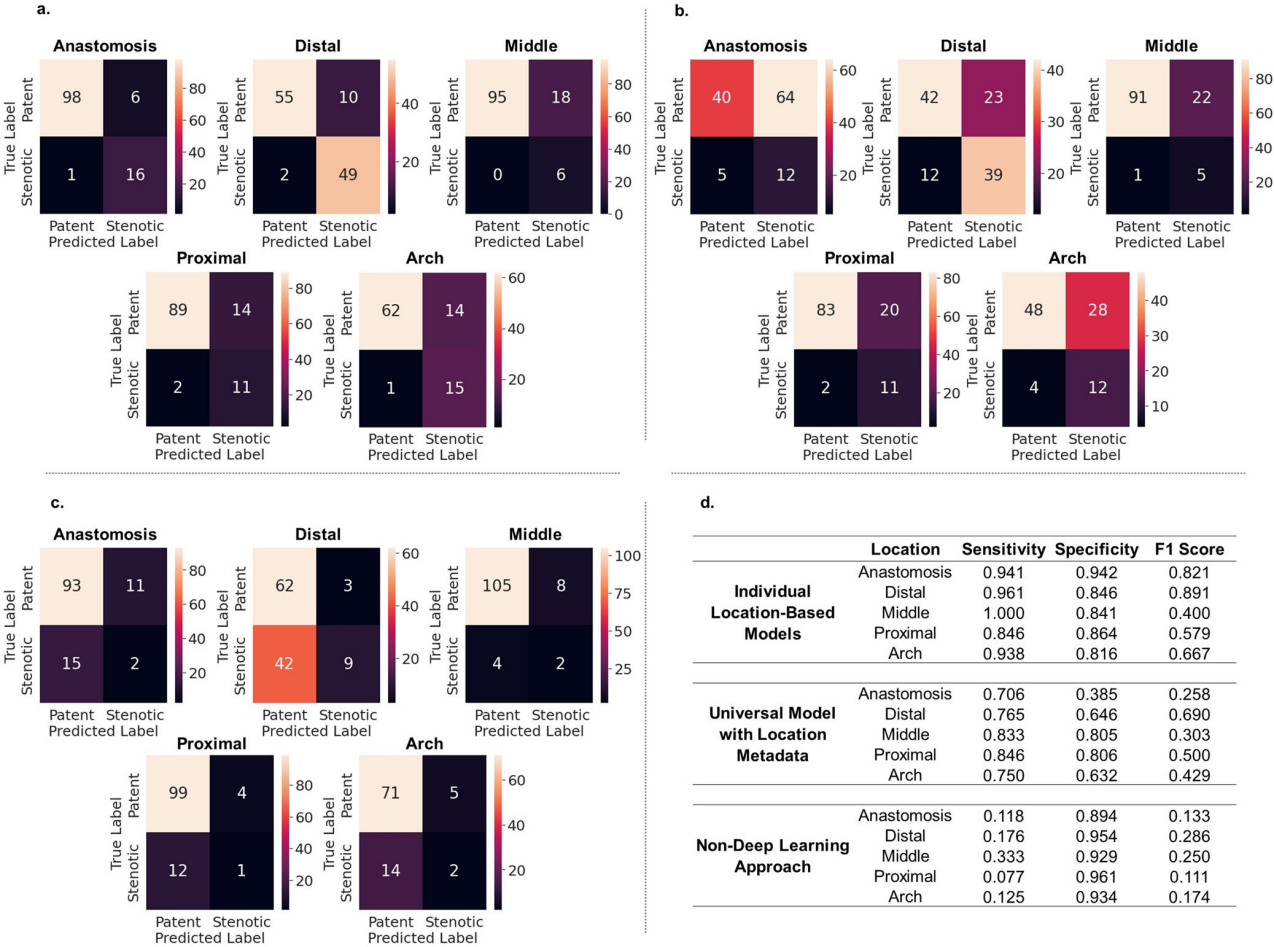
Evaluation on held-out test set. **a** Confusion matrices for the individual, location-based ViT trained on 368 × 128 spectrogram images. **b** Confusion matrices for the “universal” ViT trained 368 × 128 spectrogram images with location metadata. We stratify the results by location to allow for side-by-side comparison. **c** Confusion matrices for a simple, non-deep learning approach for detecting stenosis at each location. Here we used the averaged area under the curve (AUC) value of the averaged patent and stenotic frequency spectrums from Fig. 3 as a threshold for deciding how to classify each sound in the test set. For example, at the anastomosis site the AUC of the averaged patent frequency spectrum is 2772 and the AUC of the averaged stenotic frequency spectrum is 4142. The average of the two AUC values is 3457. In the test set, if a sound has a frequency spectrum AUC greater than 3457, we classify the sound as stenotic, and vice versa. **d** Summary of sensitivity, specificity, and F1 score for the three approaches.

Finally, we study how well our models perform on our held-out test set. In particular, we look at the individual location-based ViT models trained on 368 × 128 spectrogram images, the universal ViT model trained on 368 × 128 spectrogram images with location metadata encoded via learned embeddings, and a non-deep learning, rule-based algorithm that classifies sound based on how loud the sound is as measured by the AUC of the frequency spectrum. For the two deep learning methods, the threshold that corresponds to the largest geometric mean of sensitivity and specificity based on the averaged ROC curve from 10-fold cross-validation was selected as the final threshold value. Fig. 5 shows confusion matrixes stratified by location to allow for direct comparisons and the sensitivity, specificity and F1 scores.

### Calibration plots

**Fig. 6:**
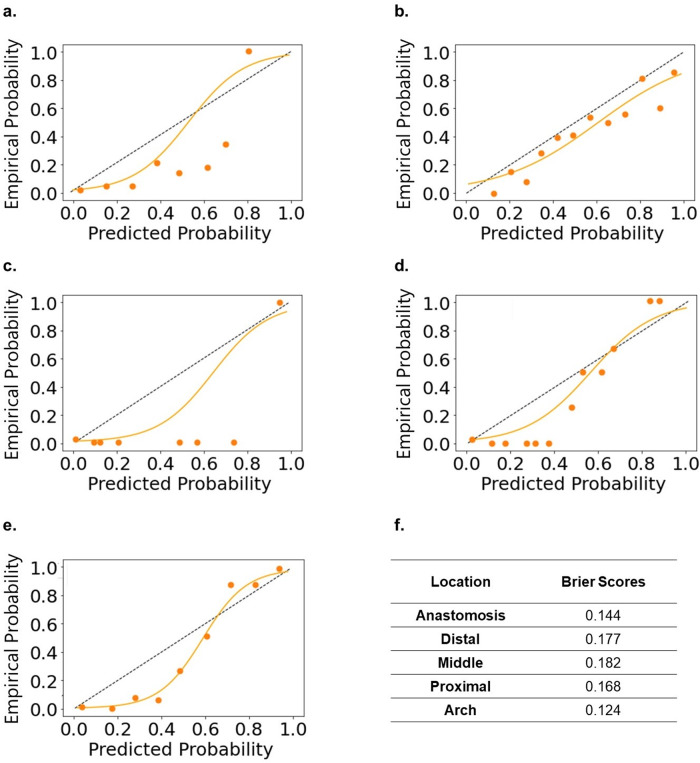
Calibration plots. Calibration plots for the individual, location-based vision transformer trained on 368 × 128 spectrogram images evaluated on the test set at each location: **a** anastomosis **b** distal **c** middle **d** proximal and **e** arch. The dotted black line represents a perfectly calibrated model. The solid orange line represents a logistic regression curve fitted to the points. **f** The Brier score for each individual, location-based vision transformer.

Fig. 6 display calibration plots for each individual, location-based ViT models trained on 368 × 128 spectrogram images along with each model’s Brier score.

### Patient level analysis

**Fig. 7:**
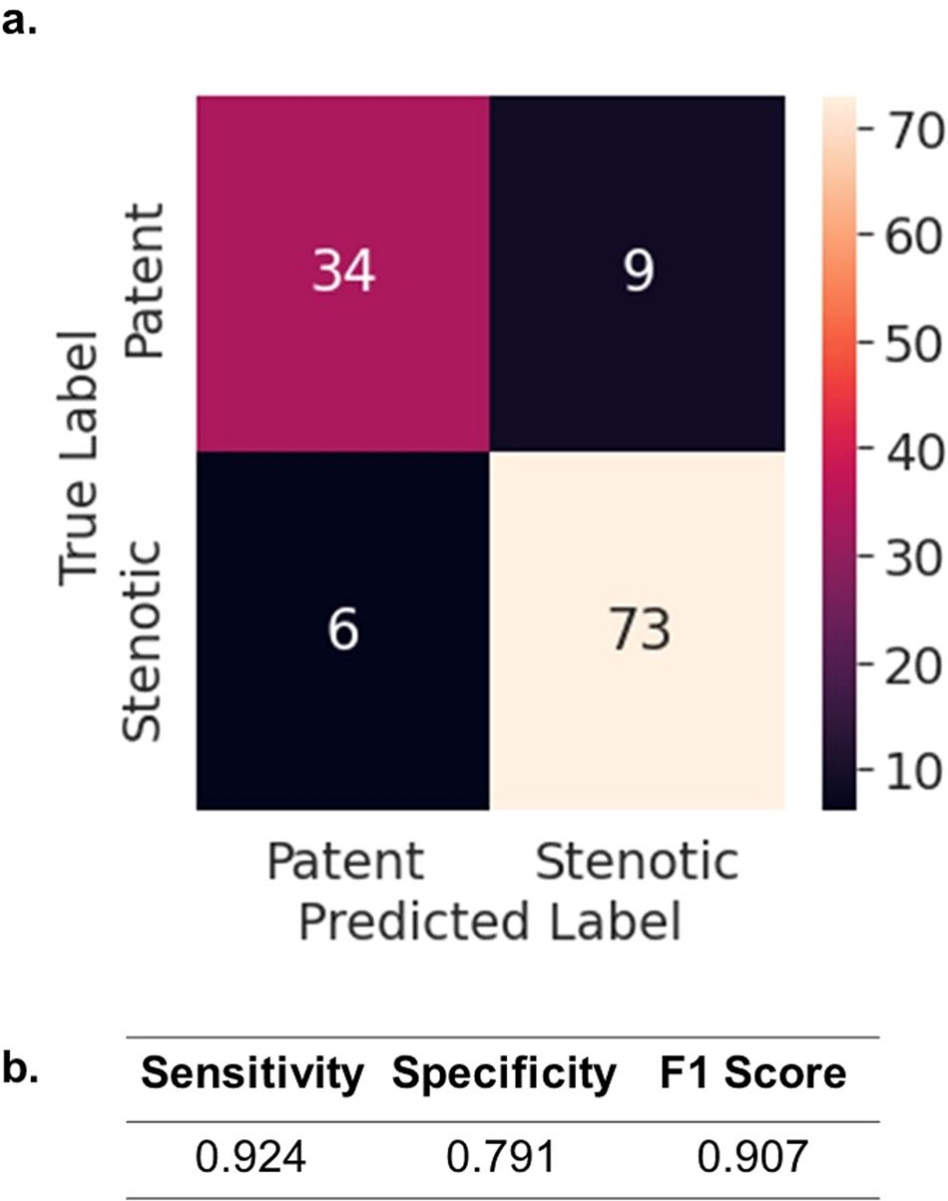
Patient level analysis. **a** Confusion matrix for the individual, location-based vision transformer trained on 368 × 128 spectrogram images evaluated on the test set at the patient level. At the patient level, the patient is considered a “stenotic patient” if the patient has a stenotic lesion anywhere along their arteriovenous fistula. If the patient has no stenotic lesions anywhere, then the patient is counted as a “patent patient”. For the predicted label for each patient, each individual, location-based model must predict patent at every location for the overall prediction to be a patent prediction. If any of the individual, location-based models predicts stenosis, then the overall prediction is counted as stenotic. **b** Sensitivity, specificity, and F1 score for the patient-level analysis.

Lastly we study how well the individual, location-based ViT models trained on 368 × 128 spectrogram images performs at the patient level. Fig. 7 shows the confusion matrix at the patient level and the sensitivity, specificity, and F1 scores.

## Discussion

Examining the frequency spectrums in our illustrative examples of stenosis at each location (Supplementary Fig. 1b–f, Supplementary Fig. 2a–f), one can see that a stenosis is characterized by a “double-peak”. The left (lower frequency peak) corresponds to diastole (when the heart’s ventricles relax) and the right (higher frequency peak) corresponds to systole (when the heart’s ventricles are contracting). During systole, there is a momentary increase in the velocity of blood flow all throughout the vasculature, including the AVF. The increased velocity through a stenosed AVF directly contributes to increasing the jet Reynolds number. The flow regime is more likely to transition to turbulent flow at the site of the stenotic lesion during systole because at baseline (during diastole) the stenotic lesions is already characterized by higher Reynolds number by virtue of the diminished lumen diameter and its direct effect on increasing velocity. This increased propensity to develop turbulent flow during systole at the stenotic site is responsible for the second higher frequency peak seen in our frequency spectrums and clinically corresponds to the “high-pitched systolic bruit of thrill” heard during auscultation. A patent AVF is better able to accommodate the increased throughput of blood during systole, and the second higher frequency peak is not as prominent or entirely absent. Supplementary Figs. 1 and 2 provides more illustrative examples of patent and frequency spectrums at each location.

To gain a better understanding of the data and to see how well these individual observations generalize, we computed the average frequency spectrum across all patients, stratified by location and patency status (Fig. 3). The “double-peaking” is not as distinct compared to the individual examples likely because the higher frequency peaks blend together when averaged. However, the distributions do appear to be bimodal, correlating with systole and diastole of the heart cycle. On average, the stenotic frequency spectrums have higher AUC values compared to their location-controlled counterparts, at all five studied locations. The AUC for the frequency spectrum corresponds to energy, which we perceive as loudness. Additionally, on average, the stenotic frequency spectrums reach higher maximum frequencies compared to the location-controlled counterparts, at all five studied locations. This is consistent with higher degrees of turbulent flow (caused by the stenosis) resulting in higher frequency components in the generated sound. Finally, on average, the stenotic frequency spectrums all have peak frequencies that are right shifted compared to the patent frequency spectrums, at all five studied locations, which correlate with the fact that even during diastole, blood is flowing faster at the stenotic site due to the reduced lumen size. In short, from our data we observe that, on average, blood flow through a stenotic lesion is louder and has higher pitch, which is consistent with the clinical physical exam^19^.

Through a series of experiments, we see if we can train a deep learning model to learn this difference in blood flow sound between a patent and stenotic AVF. In addition to the overall goal of building the best classifier, our experiments also help assess (1) how important is it to contextualize the sound with information about the location along the AVF from which the sound was sourced from and (2) how important it is to contextualize the sound with information regarding the anatomical original of the artery and vein used to construct the AVF.

Experiment one allows a direct comparison of the three different models architectures and two different pre-processing methods explored. In experiment one, we build independent classifiers trained on patent and stenotic sounds at each location, testing every combination of the three model architectures with the two pre-processing methods. The three model architectures explored are a CNN, and ResNet-50 pre-trained on ImageNet weights, and a ViT. The two pre-processing methods explored are spectrogram images and recurrence plot images.

From experiment one, we observe that spectrogram images outperform the recurrence plot image, achieving higher AuROC and AUPRC values for each model architecture (note that the AuPRC values should be interpreted in the context of the true positive rate for each location as precision and recall do not consider the true negative rate). The spectrogram images represent frequency as it varies with time, and so the spectrograms contain information from both the time and frequency domain. The recurrence plots are constructed from the frequency spectrum, and so the recurrence plots contain information only from the frequency domain. At first thought, it may be intuitive to believe that the differences between patent and stenotic sounds are only encoded in the frequency domain, as suggested by our analysis on the frequency spectrums of the sounds. However, the spectrograms outperforming the recurrence plots means there is also useful information encoded in the time domain that is helping the model learn the difference between patent and stenotic sounds. For the spectrogram images, we also explored three different time resolutions at a constant frequency resolution (374 × 128, 128 × 128, 32 × 128), and the best performing spectrogram resolution was the largest (374 × 128). Note that for the ViT, we resized the time resolution of 374 to 368 to be compatible with the 16 × 16 patch tokenization step. This further supports the argument that there are distinguishing features in the time domain and is consistent with the general idea that the model performs better when given more information to learn from. In our patient population of mature fistulas, we do not expect there to be any changes in heart rate or blood pressure based on degree of AVF stenosis and this is corroborated in Table 1. Thus, it seems unlikely that the time-dependent information being leveraged by the models is related to heart rate. We speculate that the time-domain phenomenon the models are learning is related to stenosed AVF’s having higher blood flow velocities.

From experiment one, we also observe that the vision transformer outperforms both convolutional neural network architectures on the spectrogram images. The convolution operator aggregates information via spatial sliding windows or kernels which use the same learned weights as it slides across an image. This architecture structurally introduces two important inductive biases inherent to CNN: translational equivariance and locality. Pooling layers, used in conjunction with convolutional layers in our models, helps the model achieve translational invariance. Translational equivalence and invariance mean that an object can be detected irrespective of its location in the image. The locality bias is the notion that closely space pixels are more correlated than pixels that are far away.

While spectrograms and natural images are both images from a data structure point of view (i.e. a grid of pixel values), the two images represent fundamentally different natural phenomenon. The inductive biases of translational invariance and locality structurally built into the CNN architecture are not as suitable for processing and interpreting spectrograms. While translation invariance is a good assumption for natural images whose axis convey a measure of physical distance (i.e. a cat in the upper left corner is the same as a cat in the lower right corner), the same is not true for spectrograms. A spectrogram conveys time on the x-axis and frequency on the y-axis. It may be a fair assumption that translational invariance applies to the time axis (i.e. a sound event happening at 5 s is the same as happening at 10 s), but it does not make much sense to uphold translational invariance to the frequency axis because semantic meaning is encoded in the frequency domain. Furthermore, the spectral properties of sound are non-local. The pitch of a sound is determined by the fundamental frequency, while the quality or timbre of a sound is determined by its harmonics (the nth harmonic has a frequency *F*_*n*_ = *nF*_1_, where *F*_1_ is the fundamental frequency). The fundamental frequency and its harmonics are not locally grouped despite originating from the same sound source. For example, if the fundamental frequency is 100 Hz, then its harmonics are 200 Hz, 300 Hz, etc. The locality bias, again while useful for natural images, is not a good inductive bias for spectrogram images because the frequencies associated with a given sound event are non-locally distributed.

The vision transformers, by using the self-attention mechanism, structurally lack these two inductive biases of translational invariance and locality, which are usually quite useful biases for natural images. Typically, the vision transformer must learn these inductive biases from the data itself; however, for spectrogram images it makes good sense to disregard these biases as the they do not pertain to spectrogram images. The ViT is not structurally constrained to the inductive biases of translational invariance and locality like the CNN, which allow the model to explore the parameter space more freely to find a better set of generalizable rules for classifying spectrograms. This explains the superior performance of the ViT over the convolution-based neural networks in classifying the spectrogram images of blood flow sound. Moreover, the convolution operator is a local operator, meaning only information that falls within the predefined window size can be aggregated. ViT maintain a global receptive field at every layer. Thus, ViT can learn long range dependencies and aggregate global information in early layers, resulting in improved performance^26^.

After establishing that the ViT trained with 368 × 128 spectrogram images performs the best, we use this combination to understand how important location metadata is. From qualitative inspection of the averaged frequency spectrums in Fig. 2a–e, we see how each location’s averaged frequency spectrum has a distinctive global shape, which suggests that the blood flow sounds differ from each other depending on the location. From Fig. 2f, see that at the anastomosis site, the sounds have the largest average AUC value. The sounds have the smallest average AUC value at the venous arch location. In other words, the blood flow sound is loudest at the anastomosis and softest at the venous arch, again highlighting how the characteristics of blood flow sounds changes as a function of location. Thus, it appears to be important to contextualize the blood flow sounds with location metadata.

We set out to experimentally confirm our observations through experiments I-III. In experiment I, we built independent classifiers, one for each location. In experiment II, we aggregate all the sounds from each location to train one ViT, but without any location metadata given to the model. In experiment III, we aggregate all the sounds from each location and supply location metadata to the ViT. Comparing the results between experiment II and III, we see that the AuROC and AuPRC improves from 0.68 ± 0.05 and 0.28 ± 0.09 (for the model lacking location information) to 0.82 ± 0.04 and 0.54 ± 0.08 (for the model considering location information), respectively. This jump in performance confirms the importance of accounting for the location along the AVF from which the sound was sourced from. Using learned embeddings to encode the categorical location information gave us the best performance results. Supplementary Fig. 11 shows the results for integer encoding and one-hot encoding. Interestingly, we see that using increasing scalar multiples of our integer encoding scheme (i.e. encoding “venous arch” as 1,10,100) results in progressively improved performance metrics (Supplementary Fig. 11a–c). These results are counterintuitive because in theory it should not matter what the integer values are since we are optimizing the same loss function in each case; the model can learn to increase or decrease the weights associated with location metadata and converge on the same solution. However, it seems that artificially increasing the importance of the location metadata at initialization (via larger integer values) leads to better performance. In the setting of limited data and computation resources, we speculate that increasing the importance at initialization either leads to faster convergence or helps the model escape a local minimum. The fact that we achieve progressively better results with increasing scalar integer encoding values further emphasizes the importance of contextualizing the sounds with location metadata.

Next, we seek to understand if it is important to contextualize the blood flow sound with metadata regarding the anatomical original of the artery and vein used in the creation of the AVF. In this study we used AVFs made from the brachial and radial artery, and the cephalic and basilic vein. In experiment IV, we test if a ViT can distinguish the brachial from the radial artery based on blood flow collected at the “artery” location. Results are shown in Supplementary Fig. 12. An AuROC value of 0.78 ± 0.11 suggest that there is a difference in blood flow sound between the radial from brachial artery. The difference in sound likely stems from the fact that the brachial artery is almost two times larger than the radial artery and has thicker vessel walls^27,28^. In experiment V, we test if a ViT can distinguish the cephalic from the basilic vein based on blood flow collected at the “arch” location. Results are shown in Supplementary Fig. 13. An AuROC value of 0.52 ± 0.13 suggest that there is not much difference in blood flow sound between a cephalic and basilic vein. The difference between the basilic and cephalic vein is only about 1–2 mm in most people, which likely explains the model’s lack of ability to differentiate the sound of blood flow between the veins^29,30^. In experiment VI, we test how well the individual, location-based ViTs perform when also given metadata regarding the anatomical origin of either the artery or the vein. We notice no improvement between the models given venous origin metadata in experiment VI compared with the models in experiment I (Supplementary Table 1), consistent with our model’s lack of ability to discern cephalic from basilic vein in experiment V. Interestingly, despite our model being able to distinguish the radial from the brachial artery, there is no improvement between the models given artery origin information in experiment VI compared with the models in experiment I (Supplementary Table 1). Thus, the anatomical original of the artery or vein seems to be unimportant in the context of building classifiers to identify AVF stenoses based on blood flow sound.

On evaluation on the held-out test set, we see that the individual, location-based ViTs outperform the universal ViT with location metadata (Fig. 5a, b). The individual, location-based models implicitly contextualize the sounds with location information since they are only trained on sounds coming from the given location. The individual, location-based ViTs can focus exclusively on learning the features that distinguish patent from stenotic at that given location. The “universal” ViT must learn a feature extractor that generalizes across all six locations, which likely hinders performance because the relevant features that define patent vs stenotic varies with location due to inherent differences in sound at each location. What it means to be “stenotic” at the “arch” location is different than “stenotic” at the “anastomosis” location, despite both receiving the same “stenotic” label. We can qualitatively see these differences in Fig. 3a, e. For example, on average, the blood flow sound is louder at a patent anastomosis site compared to a stenotic venous arch site.

In evaluation on our test set, we also tested a simple non-deep learning approach based on our conclusion that, on average, the blood flow through stenotic lesions is louder than through patent vessels (Fig. 3). For each location, the half-way point between the averaged patent frequency spectrum AUC value and the averaged stenotic frequency spectrum AUC value is used as a threshold for evaluating the test set. For the test set, sounds with frequency spectrums AUC values that fall above the threshold are classified as stenotic, and those with AUC values below the threshold are classified as patent. This approach gives us inferior results compared to the two deep learning approaches. While general spectral properties that correlate clinically seem to emerge from the averaged frequency spectrums, judging from both the large standard deviations in Fig. 2f and from visual inspection of the individual frequency spectrums in Supplementary Figs. 1 and 2, there seems to be large degree of heterogeneity among the sounds on an individual level. This underscores the need for highly parameterized deep learning models over simpler rule-based algorithms for screening for AVF stenosis based on blood flow sound. Finally, we perform a patient-level analysis on our held-out test set using our best performing model, and we achieve a sensitivity, specificity, and F1 score of 0.924, 0.791, 0.907, respectively (Fig. 6). As a reference for performance, a clinical trial that studied how well a single expert nephrologist could identify stenosis in hemodialysis arteriovenous fistulas based on a physical exam, also using ultrasound as the ground truth, reported a sensitivity of 0.96 and a specificity of 0.76^31^. Thus, our model is able to screen for stenosis at a level comparable to that of an expert nephrologist performing a physical exam.

One of the limitations of this study is that we only studied brachial/radial – cephalic/basilic fistulas. Although the most common types of fistulas, other fistula types using other artery and veins exist, and our conclusion that the anatomical origin of the artery and vein is not important may not generalize. Additionally, our model cannot be used to identify stenosis on the arterial side of an AVF, although this is much rarer than stenosis on the venous side. This is due to the lack of training data we have of arterial stenosis (only 6 examples). Furthermore, an important clinical implication of adjusting for class imbalance during our training process is that this can potentially cause the model to be mis-calibrated. In our case, we are using a weighted loss function (in essence, oversampling the minority class), which can potentially cause the model to be overconfident when making positive class (i.e. stenotic sounds) predictions. Empirically from our calibration plots for the individual, location-based ViT shown in Fig. 6, we do see that our model tends to be overconfident, which is another limitation for our model. While overconfident predictions may be the result of our class imbalance adjustments, we find our class imbalance adjustments necessary to achieve good model discrimination. We show the ROC and PR curves from 10-fold cross-validation for the ViT trained on 368x128 spectrogram images without using a weighted loss function in Supplementary Fig. 14. Compared to the ROC and PR curves shown in Fig. 3c, we can see how adjusting for class imbalance improves model discrimination in our case. Another important limitation of this study is how we validated our data. Stenotic lesions were identified with duplex ultrasound. Clinically, a stenotic lesion identified on ultrasound does not always necessitate a percutaneous angioplasty (the procedure for treating a stenotic AVF). An important clinical question is when to intervene on a stenotic AVF once found. While our study demonstrates promise for using deep learning analysis on blood flow sound as a quick and economical screening tool for identifying the presence of stenotic lesions, future work correlating sound to AVFs that ultimately require percutaneous angioplasties may further improve the utility of such technology.

In summary, our study presents a novel, fast, and easy approach for screening for AVF stenosis in hemodialysis patients using deep learning to analyze the sound of AVF blood flow. The final models we recommend for deployment are the individual, location-based vision transformer models trained on 368 × 128 spectrogram images. Our preliminary model evaluation shows that this technology can screen for stenosis at a level comparable to that of a nephrologist performing the physical exam, but with the advantage of being automated and scalable. In routine practice, the onus of performing the physical exam to screen for stenosis during dialysis sessions typically falls on the dialysis technician. Thus, this technology could help dialysis technicians, who are often challenged with a high-volume of patients each day, ensure patient safety while also streamlining workflows to reduce costs. The clinical implication is that our new screening tool can help catch cases of stenosis that may otherwise be missed due to understaffed dialysis centers (the patient to staff ratios at dialysis centers can exceed 90:1 and reach upwards of 300% the recommended limit by the NKF)^32,33^. Additionally, our technology could serve as an indirect gateway to ultrasound in the diagnostic workup. Instead of performing an ultrasound on every patient, routine screening can be done via our technology and screening ultrasound is only performed on those flagged for potential stenosis to help facilitate efficient resource allocation. Note that routine ultrasound screening is separate from the routine physical exam screening that is to be performed at each dialysis session. We foresee our technology facilitating the screening process that takes place at the dialysis sessions, and not to be used as a complete replacement for ultrasound. There is potential for this technology to even be patient facing. The next step in implementation would be to deploy the model onto a server and create an API that will allow users to upload a sound and receive back a prediction. The next step in terms of validation of effectiveness and regulation would be to run a prospective clinical trial using our deployed model.

## Methods

### Turbulence induced sound

The sound produced by blood flowing through an AVF can be an important indicator of the AVF’s patency status. Blood flow through a patent AVF is laminar and will create a quiet “whooshing” sound. A stenosed AVF can be conceptualized as a converging-diverging nozzle. Flow through a converging-diverging nozzle is characterized by jet Reynolds number show in Eq. 1:

1 $$\mathrm{Re}=\frac{{uD}}{v}$$

where *u* is the velocity, *D* is the jet diameter, *v* is the kinematic viscosity of the fluid. Experiments have shown that if *Re* exceeds about 2000, the jet flow will be turbulent^34^. A stenosed AVF will have a reduced lumen diameter relative to a patent AVF. By conservation of mass and momentum, as the lumen diameter decreases, fluid velocity will increase. From the jet Reynolds equation, we can see that this inherent inverse relationship between velocity and diameter means that velocity and diameter have opposing effects in determining the overall Reynolds number. However, as an AVF develops stenosis, the velocity of blood flow will increase by a larger factor relative to how much the diameter will decrease. This can be understood from a simplified volumetric flow rate equation Q= ${u}_{1}\left(\pi {r}_{1}^{2}\right)={u}_{2}\left(\pi {r}_{2}^{2}\right)$, where Q is the constant volumetric flow rate, *u*_1_ is the fluid velocity at radius *r*_1_ and *u*_2_ is the fluid velocity at radius *r*_2_, assuming an incompressible, Newtonian fluid, which is an acceptable assumption for blood^35^. In this simplified model, a reduction in the lumen radius by 2 will result in an increase in velocity by a factor of 4. In other words, as an AVF develops stenosis, the increased fluid velocity *u* caused by the reduced diameter *D* will overall result in a net increase of the jet Reynolds number. Once the jet Reynolds number crosses a certain threshold (i.e. 2000), the flow regime will transition from laminar to turbulent. Turbulent flow produces a different sound compared to laminar flow. This concept of turbulent fluid induced noise is characterized by Lighthill’s wave equation. Turbulent fluid flow collaterally generates pressure and density variations in the fluid, which in turn generates the pressure and density variations that we perceive as noise^36^. Increasing turbulence will result in an increased amount of higher frequency components in the generated sound^37^. Clinically, the sound heard when auscultating a stenosed AVF is often described as a “high-pitched systolic bruit or thrill” (Fig. 1c).

### Data collection

A total of 433 patients with AVFs were enrolled in this study. All recordings were performed in the same clinical setting, which is an outpatient vascular ultrasound lab. The enrolled patients are visiting clinic for routine ultrasound screening. Patients with AVFs post-ESRD and pre-ESRD (pre-emptively placed AVF in light of deteriorating kidney function) were included in this study. Patients with arteriovenous fistulas, created with either the radial or brachial artery and either the cephalic or basilic vein, were recruited for this study. On the arterial side, 80% of patients had fistulas created from the brachial artery; 20% of patients had fistulas created from the radial artery. On the venous side, 65% of patients had fistulas created from the cephalic vein, 35% of patients had fistulas created from the basilic vein. In summary, four fistula variations are analyzed in this study: brachiocephalic fistulas (52%), brachiobasilic fistulas (28%), radiocephalic fistulas (13%), radiobasilic fistulas (7%).

For each patient, blood flow sounds were collected at 6 different locations along the patient’s AVF (Fig. 1b). Of the 6 sounds, one was collected from the artery, one was collected at the anastomosis site (i.e., where the artery has been surgically joined to the vein), and four sounds were collected along the vein. The locations were designated, from most distal to most proximal, as “arterial” for the artery, “anastomosis” for the anastomosis site, “distal” for the distal vein, “middle” for the middle vein, “proximal” for the proximal vein, and “arch” for the arch of the vein (i.e., the point along the fistula closest to the shoulder). Note we use terminology “proximal” and “distal” based on the anatomic definitions of the arm. A total of 2565 AVF blood flow sounds were included in this study. Sounds were collected using a 3 M Littmann Core digital stethoscope at a sampling rate of 4000 Hz. Each sound was recorded for 15 s. Sounds were collected over a two year period from 2021 to 2023.

The sounds from the blood flow were labeled as “patent” (normal) or “stenotic” (abnormal). The labels are validated from concurrent duplex ultrasound (blood flow sound recorded by stethoscope and ultrasound imaging were done at the same time). The final label of “patent” vs “stenotic” at each location was determined after interpretation of the corresponding ultrasound imaging and velocity reports by a board-certified vascular surgeon. The diagnosis of stenosis is established when the measured blood flow velocity by duplex ultrasound is at least double that of a preceding segment. Our dataset included 2113 patent sounds (83%) and 452 stenotic sounds (17%). Note that for some patients only 5 sounds were collected. Instead of discarding an “incomplete” set, we kept them in the study to maximize the number of samples.

The data was divided into train, validate, and test sets. First, 20% of the data was randomly reserved to serve as the held-out test set for final model evaluation. Then 10-fold cross-validation was used within the training dataset (the remaining 80%). Cross-validation is used throughout the experiments (explained in more detailed below) for model training, model hyperparameter tuning and optimization, and comparison among models. The splits are done on the patient-level to prevent data leakage. Of the patients that do have a stenotic lesion, the vast majority will only have 1 stenotic lesion. There are a few cases where a patient has a stenotic lesion present at 2 separate sites; however, since the train, validation, and testing splits were done on the patient level, they would both appear in the same set.

### Deep learning models

Three different deep learning models were explored in this study: a convolutional neural network (CNN) trained with no preset weights, a ResNet-50 pre-trained on ImageNet, and a vision transformer (ViT) with no preset weights. The CNN consisted of 6 convolutional layers. The number of filters used was 8, 16, 32, 64, 128, 256 for the 1st, 2nd, 3rd, 4th, 5th, 6th layer, respectively. Each layer uses a rectified linear (ReLu) activation function. Following each convolutional layer was a max pooling and batch normalization layer. After the six convolutional layers, the feature vector is flattened via global average pooling. The feature vector is then fed into three fully connected layers consisting of 32, 16, and 1 node(s). The first two fully connected layers uses a ReLu activation function, while the last node uses a sigmoid activation function to perform the final binary classification of “Patent” versus “Stenotic”. This model was trained using an adaptive moment estimation (Adam) optimizer at a learning rate of 1 × 10^−3^. To address the issue of class imbalance, a weighted binary cross-entropy loss function which gives more importance to the minority class (i.e., the stenotic sounds) is used to calculate the loss. The class weights ratio used mirror the inverse of the class distribution in the training set. The same weighted binary cross-entropy loss function is used with the other models as well. An illustration of the 6-layer CNN is shown (Fig. 4a).

The second model explored was a ResNet-50. In brief, a ResNet-50 is a CNN that is 50 layers deep with residual or skip connections that allows activations from earlier layers to be propagated down to deeper layers^38^. For this model, we also leverage transfer learning by using a ResNet-50 pre-trained on ImageNet21k, a large dataset consisting of over 14 million natural images that belong to over 20,000 classes^39^. One fully connected layer consisting of one node with a sigmoid activation function was added on top of the ResNet-50 to perform the final binary classification of “Patent” versus “Stenotic”. This model was trained using an Adam optimizer over the weighted binary cross-entropy loss function. First, the ResNet-50 weights were kept frozen only the final fully connected layer was trained at a learning rate of 1 × 10^−3^. Then the entire model (ResNet-50 plus the fully connected layer) was finetuned, trained at a learning rate of 1 × 10^−5^. An illustration of the ResNet-50 is shown (Fig. 4a).

The final model explored was a ViT. For our ViT, first the model input is tokenized into 16 × 16 patches. The patches are flattened and fed into a linear transformation layer to create a lower dimensional embedding and combined with positional encodings, which are learnable embeddings. The embedded patches are then inputted into a sequence of 10 transformer encoders. Each transformer encoder is comprised from 2 subcomponents. For each encoder, the first subcomponent is a 6-headed multi-attention layer, which implements the multi-headed self-attention mechanism. The second subcomponent for each encoder is a fully connected feed-forward network using ReLu activation functions. After the 10 transformer encoders, the feature vector is flattened and passed to 3 fully connected layers consisting of 2048, 1024, and 1 node(s). The first two fully connected layers uses a ReLu activation function, while the last node uses a sigmoid activation function to perform the final binary classification of patent versus stenotic. This model was trained using an adaptive moment estimation (Adam) optimizer at a learning rate of 1 × 10^−3^ over the weighted binary cross-entropy loss function. An illustration of the ViT in shown (Fig. 4a). All models are trained for 200 epochs, and the weights that correspond to the lowest validation loss are take to be the final model weights.

### Pre-processing

Our three chosen models work with two-dimensional image data, while our raw audio data is one-dimensional timeseries data. To make our data compatible with our models, we first preprocess our audio data into two-dimensional image representations. Two different image representations of sound are explored in this study: Mel-scaled, decibel (dB)-scaled spectrograms and recurrence plots.

A spectrogram depicts the spectrum of frequencies of a signal as it varies with time. The x-axis represents time, the y-axis represents frequency, and amplitude of a particular frequency component at a given point in time is represented by the intensity of color. The spectrograms are generated from the AVF blood flow sounds using short-time Fourier transforms as follows. First, the audio signals are windowed using a Hann window of size 512 and a hop length of 256. A 512-point fast Fourier transform is applied to each window to generate a spectrogram. The Mel-scaled, dB-scaled spectrograms are generated by logarithmic rescaling of the amplitude and frequency axis. The amplitude axis is converted to the dB scale. The frequency axis is transformed onto the Mel scale, characterized by Eq. 2,

2 $${Mel}=2595* {\rm{log }}\left(1+\frac{f}{500}\right)$$

where *f* is frequency in Hz. The resulting Mel-scaled, dB-scaled spectrograms are 374 × 128 (time resolution x frequency resolution) in size. To study the effects of varying time resolution on the spectrogram image, spectrograms with dimensions 128 × 128 and 32 × 128 are also created using bicubic interpolation. The time domain encompasses 15 s.

A recurrence plot is an image that visualizes the set of all pairs in time (*t*_*n*_*, t*_*m*_) in which $\vec{x}\left({t}_{n}\right)=\vec{x}\left({t}_{m}\right),$ where $\vec{x}$ is the systems trajectory vector through the phase space. The phase space is a multidimensional space that represents every possible state of a system, with each degree of freedom of a system represented as an axis^40^. In this study, we generate recurrence plots of the frequency spectrum. First, a Fourier transform is applied over the entire audio signal to generate the frequency spectrum. Then the frequency spectrum is discretized. For example, let $T=\{{t}_{0},{t}_{1},{t}_{2},\ldots {t}_{n}\ldots {t}_{N}$ represent the discretized points over which the frequency spectrum spans, separated by the interval δ. Then the trajectory of the frequency spectrum through the phase space is given by $\vec{X}=\{{\vec{x}(t}_{0}),{\vec{x}(t}_{1}),{\vec{x}(t}_{2}),\ldots {\vec{x}(t}_{n})\ldots {\vec{x}(t}_{N})$. The recurrence states of $\vec{x}\left({t}_{n}\right)$ are states $\vec{x}\left({t}_{m}\right)$ that fall within a given radius Ɛ around $\vec{x}\left({t}_{n}\right)$. The recurrence plot is constructed as an *N x N* lattice of squares with side length δ and with each coordinate axis reporting T. The value at coordinates $\left({t}_{n},{t}_{m}\right)$ is given by the recurrence value function ${\rm{R}}({t}_{n},{t}_{m})=\Theta ({\rm{\varepsilon }}-{||}\vec{x}\left({t}_{n}\right)-\vec{x}\left({t}_{m}\right){||})$, where $\Theta$ is the Heaviside step function. The final recurrence plots are size 128 × 128. All images representations (both recurrence plots and spectrograms) are normalized prior to input into the model into the range [−1,1].

### Averaged frequency spectrums

An averaged frequency spectrum is computed across all patients in the train and validate sets, stratified by label and location. Four spectral parameters are extracted from each frequency spectrum: total area under the curve (AUC), peak frequency, max frequency, and full width at half max (FWHM). The frequency spectrum is used to extract four spectral parameters from each AVF recording. Total area under the curve (AUC) is approximated using the composite trapezoidal rule for definite integrals, defined as $${\int }_{a}^{b}f\left(x\right){dx}=\frac{1}{2}\mathop{\sum }\nolimits_{j=1}^{n}\left({x}_{j}-{x}_{j-1}\right)[f\left({x}_{j}\right)+f({x}_{j-1})]$$, with partition length of 0.1 i.e., $${x}_{j}-{x}_{j-1}=0.1)$$ and frequency range (a–b) of 0–2000 Hz. Peak frequency ($${x}_{{peak}}$$) is defined as the frequency value that corresponds to the peak of the highest amplitude. Maximum frequency is estimated as the highest frequency with amplitude greater than 0.1. Full width at half max (FWHM) is calculated using the horizontal frequency span at half of the maximum amplitude, where $${FWHM}={x}_{n}-{x}_{m}$$, and $$f\left({x}_{n}\right)=f\left({x}_{m}\right)=\frac{1}{2}f({x}_{{{\rm{peak}}}})$$.

A simple, non-deep learning approach is explored using the AUC values from the averaged frequency spectrums. For each location, the half-way point between the averaged patent frequency spectrum AUC value and the averaged stenotic frequency spectrum AUC value is used as a threshold for evaluating the test set. For the test set, frequency spectrums AUC values that fall above the threshold are classified as stenotic, and those with AUC values below the threshold are classified as patent.

## Experiments

In experiment I, we build independent, location-based binary classifiers, one for each of the following locations: “anastomosis”, “distal”, “middle”, proximal”, and “arch”. In other words, each location-based model is trained only on sounds originating at the given location. Note we do not build a model for the arterial location given we only have 6 examples of stenosis. For each location, we test the three different model architectures (a 6-layer CNN, a ResNet-50 pre-trained on ImageNet weights, and a ViT) with the two pre-processing methods (spectrograms and recurrence plot images). For the spectrogram images, we tested 3 different sizes of varying time resolution at the constant, maximum frequency resolution of 128: 374 × 128, 128 × 128, and 32 × 128. Note that for the ViT, the 374 × 128 spectrogram image is resized to be 368 × 128 to be compatible with the 16 × 16 patch tokenization step.

In experiment II, we test how well a ViT trained on 368 × 128 spectrogram images performs in classifying the blood flow audio signal as patent or stenotic using audio signals from all six locations, but without supplying the model with any metadata regarding which location the sound is sourced from.

In experiment III, we test how well a ViT trained on 368 × 128 spectrogram images performs in classifying the blood flow audio signal as patent or stenotic using audio signals from all six locations, this time with location metadata regarding where the sound is being sourced from explicitly fed into the model. This is accomplished by first encoding the categorical location information into some numerical representation, and then concatenating that numerical representation to the feature vector coming from the last transformer encoder layer. We explore three different methods of encoding the categorical location metadata: an ordinal encoding scheme where each location is encoded as an integer, using one-hot encoding, and using a learned embedding. For the learned embedding layer, a 6 × 4 embedding matrix *E* is learned as part of the training. Within the ordinal encoding scheme, we study the effects of using scalar multiples of the integer encodings. An illustration of this modified ViT architecture is shown in Fig. 4a.

In experiment IV, we test if we can build a binary classifier to distinguish if the blood flow audio signal is coming from either the radial or brachial artery. For this task, we train the ViT on spectrogram images using only patent radial and patent brachial sounds taken at the “artery” location.

In experiment V, we test if we can build a binary classifier to distinguish if the blood flow audio signal is coming from either the basilic or cephalic vein. For this task, we train the ViT on spectrogram images using only patent cephalic and patent basilic sounds taken at the “arch” location.

In experiment VI, we test how well a ViT trained on 368x128 spectrogram images performs in classifying the blood flow audio signals as patent or stenotic when also given information about the anatomical original of either the artery or vein used in the creation of the fistula, for each location. This is accomplished in a parallel manner to experiment III, where first the categorical information about the anatomical origin of the artery or vein is encoded as different integers (1 for brachial artery, 0 for radial artery; 1 for cephalic vein, 0 for basilic vein), and then concatenated to the feature vector coming from the last transformer encoder layer. An illustration of this modified ViT architecture is shown in Fig. 4a.

### Supplementary information

SUPPLEMENTAL MATERIAL

## Data Availability

The data that support the findings of this study are available from the corresponding author upon reasonable request.

## Code availability

The code that support the findings of this study are available from the corresponding author upon reasonable request.
