# Supplementary material for: Deep learning analysis of blood flow sounds to detect arteriovenous fistula stenosis
Source: NPJ Digit Med. 2023 Sep 1;6:163. doi: 10.1038/s41746-023-00894-9 (PMC10474109; doi:10.1038/s41746-023-00894-9)
Supplement: Supplementary file 1 — SUPPLEMENTAL MATERIAL [file 41746_2023_894_MOESM1_ESM.pdf]

Supplementary Information

Supplementary Figure 1. Illustrative examples of data Blood flow waveforms, frequency spectrums, mel-scaled, db-scaled spectrogram images and recurrence plot images are shown at all six locations from **a)** a patient with an entirely patent arteriovenous fistula, **b)** a patient with an arteriovenous fistula with a stenotic lesion at the anastomosis site, **c)** a patient with an arteriovenous fistula with a stenotic lesion at the distal vein site, **d)** a patient with an arteriovenous fistula with a stenotic lesion at the middle vein site, **e)** a patient with an arteriovenous fistula with a stenotic lesion at the proximal vein site, **f)** a patient with an arteriovenous fistula with a stenotic lesion at the venous arch site. The stenotic lesions are indicated in red.

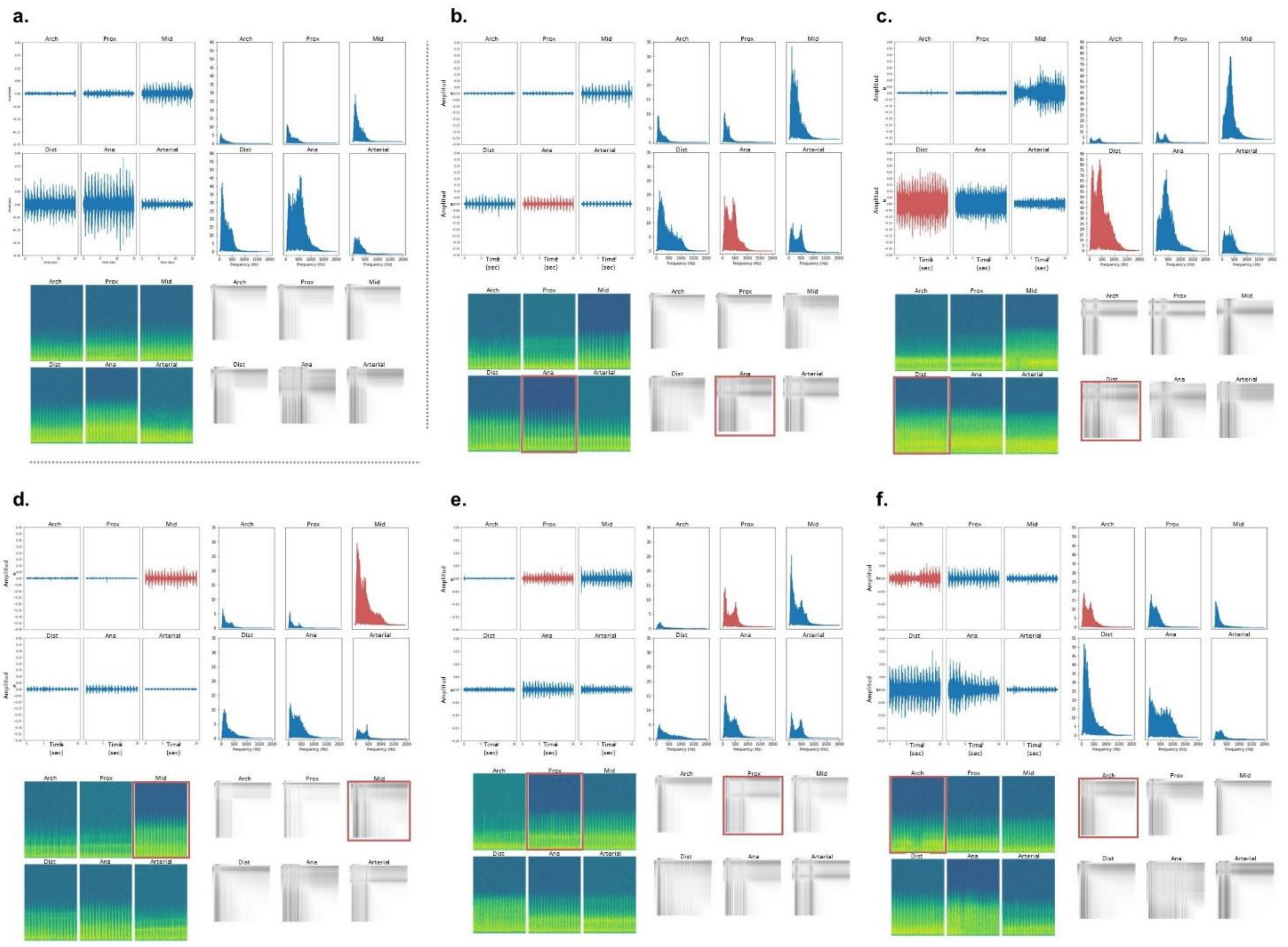

Supplementary Figure 2. Additional illustrative examples of patent (blue) and stenotic (red) frequency spectrums at each location **a)** Anastomosis **b)** Distal **c)** Middle **d)** Proximal **e)** Arch

a)

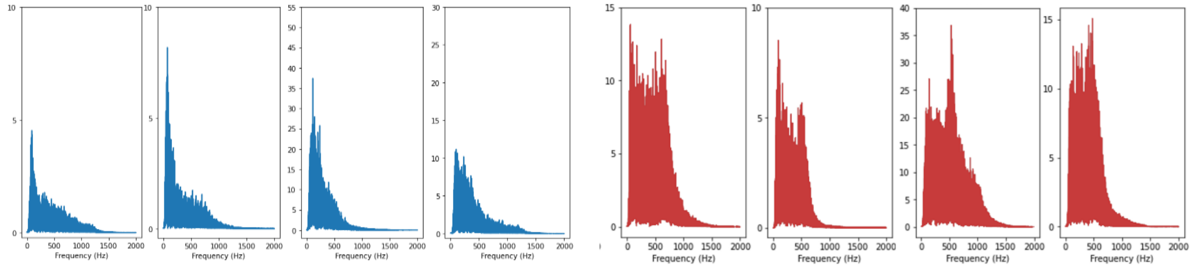

b)

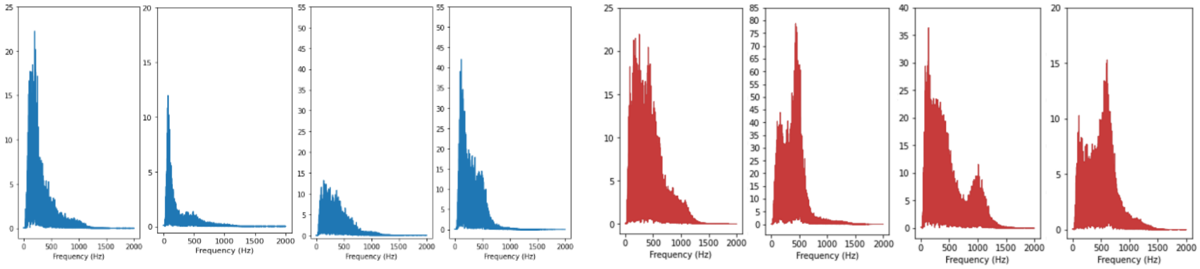

c)

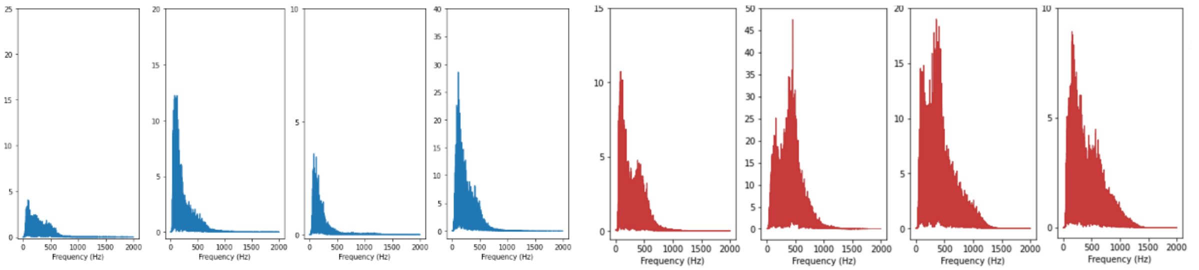

d)

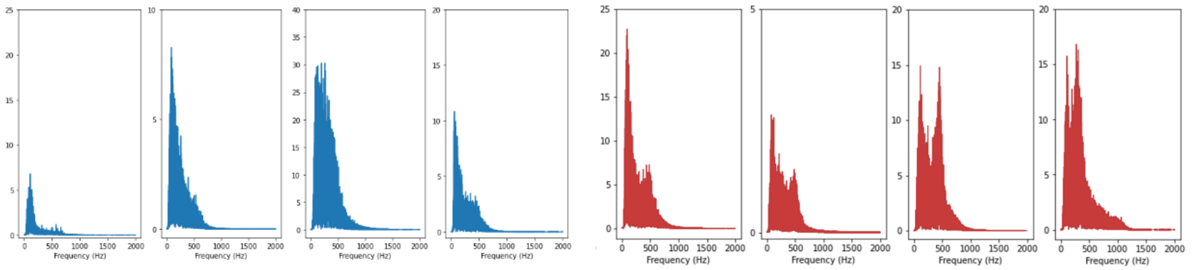

e)

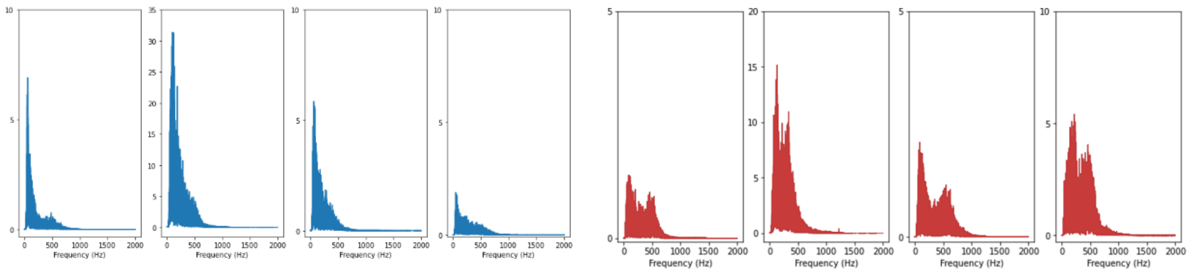

Supplementary Figure 3. The ROC (left) and PR curves (right) from 10-fold cross validation for the conventional CNN trained on 374x128 spectrogram images for each location **a)** Anastomosis **b)** Distal **c)** Middle **d)** Proximal **e)** Arch

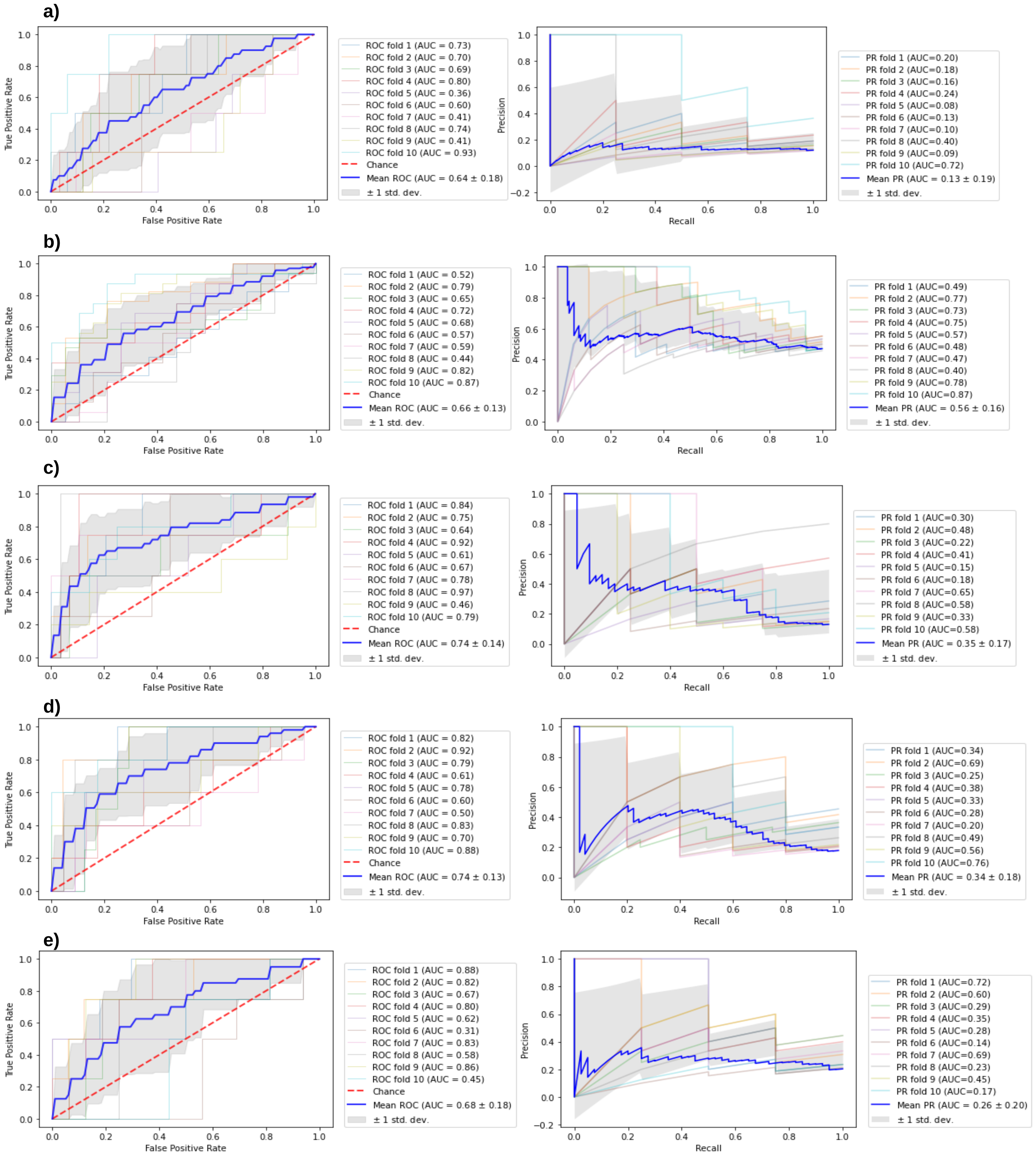

Supplementary Figure 4. The ROC (left) and PR curves (right) from 10-fold cross validation for the conventional CNN trained on 128x128 recurrence plots for each location **a)** Anastomosis **b)** Distal **c)** Middle **d)** Proximal **e)** Arch

a)

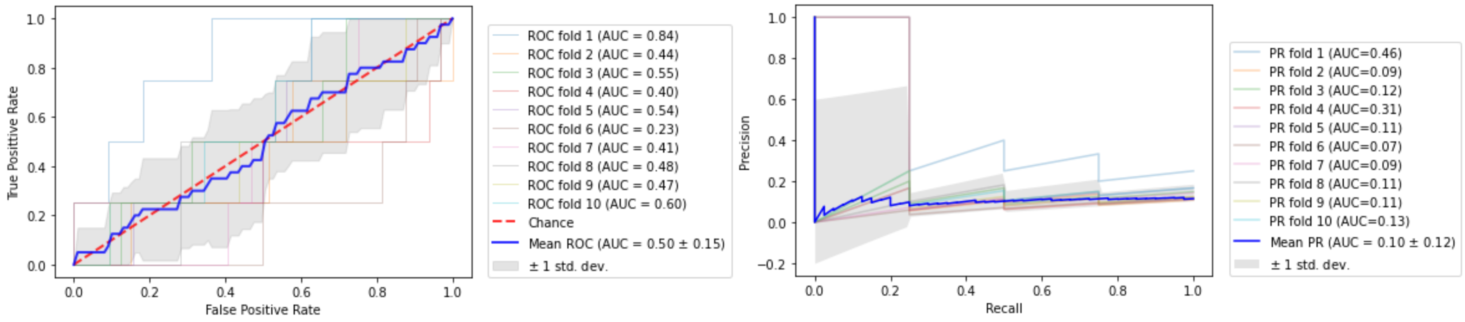

b)

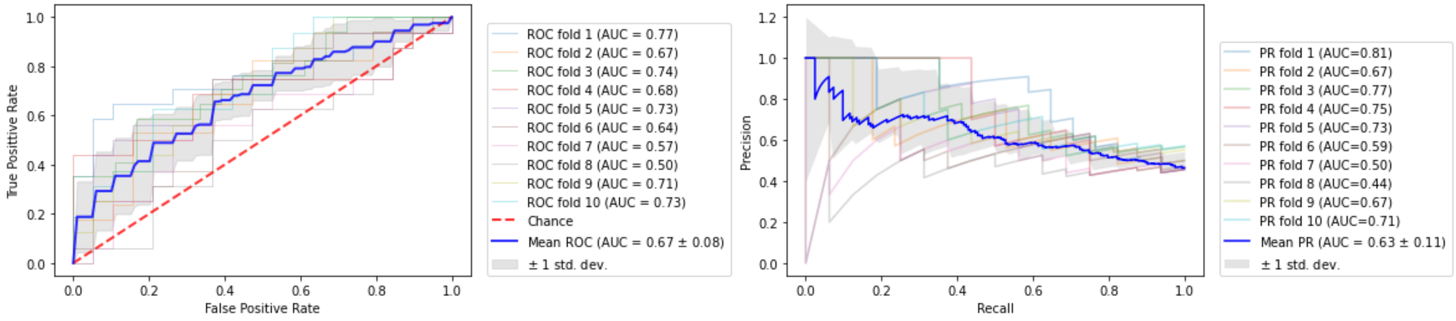

c)

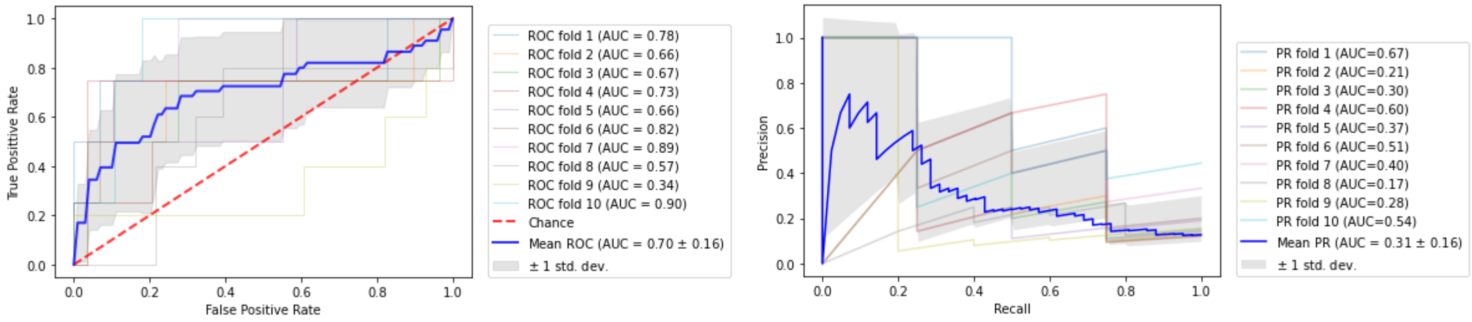

d)

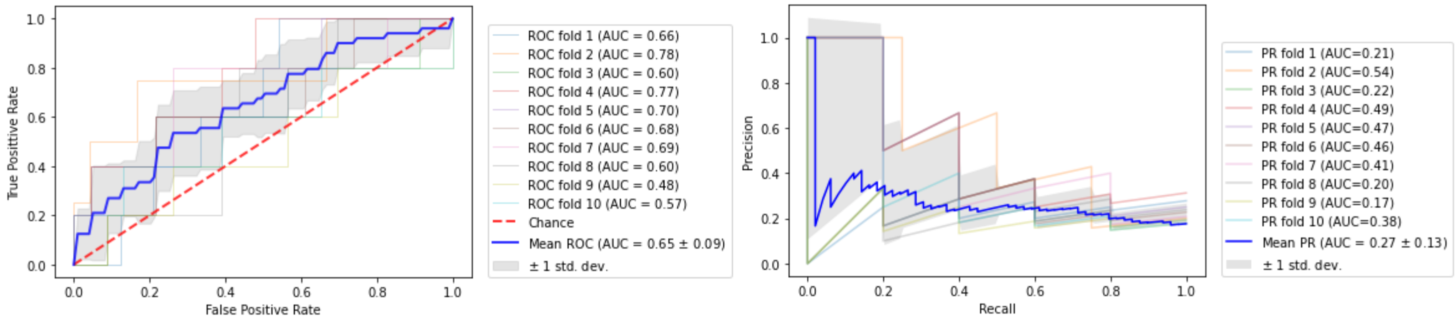

e)

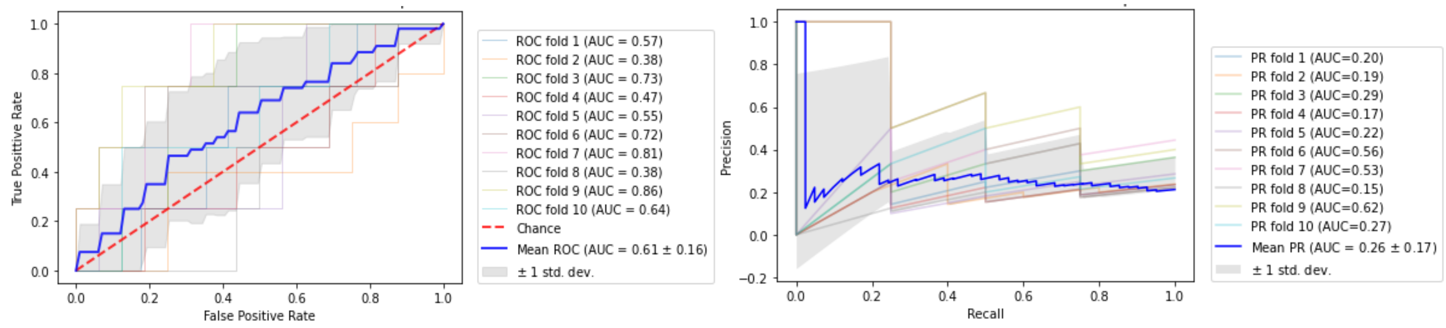

Supplementary Figure 5. The ROC (left) and PR curves (right) from 10-fold cross validation for the ResNet-50 trained on 374x128 spectrogram images for each location **a)** Anastomosis **b)** Distal **c)** Middle **d)** Proximal **e)** Arch

a)

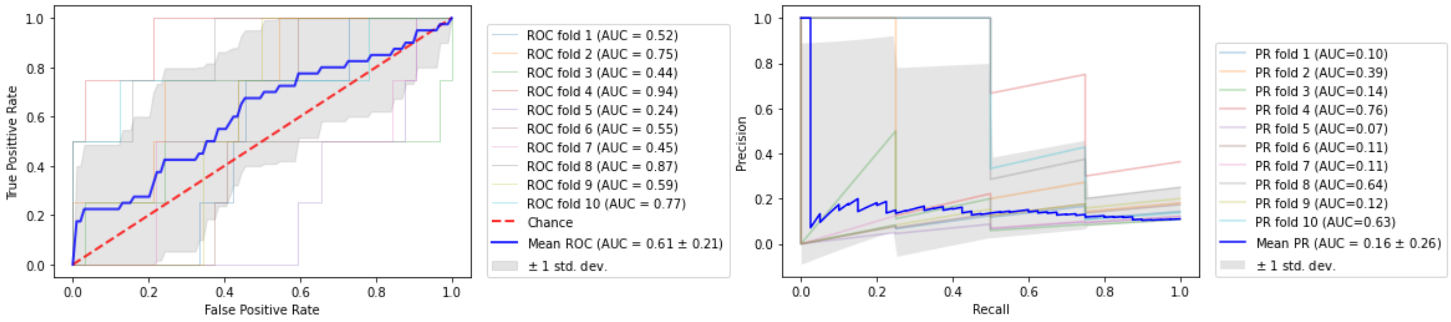

b)

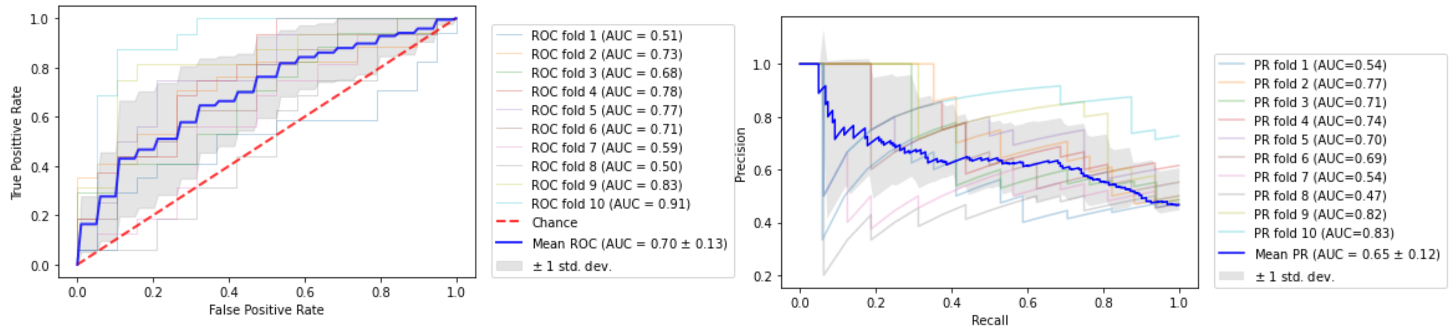

c)

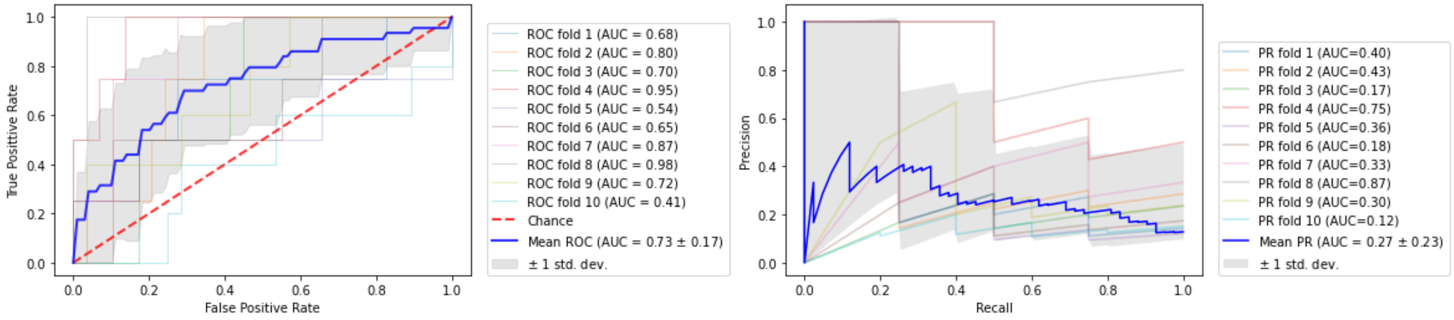

d)

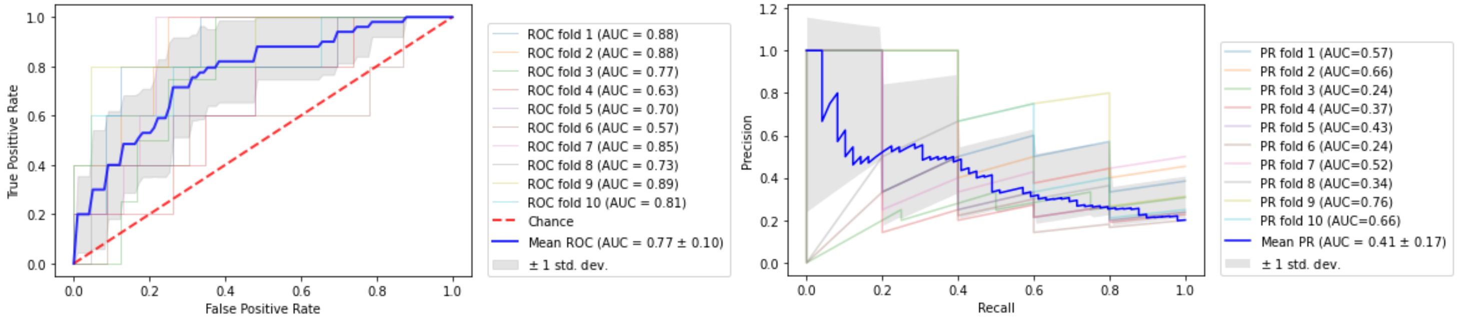

e)

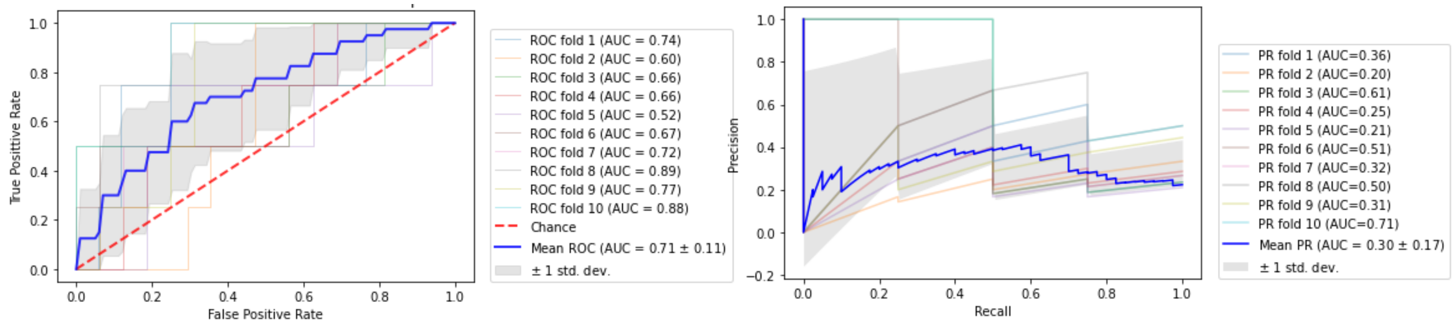

Supplementary Figure 6. The ROC (left) and PR curves (right) from 10-fold cross validation for the ResNet-50 trained on 128x128 recurrence plots for each location **a)** Anastomosis **b)** Distal **c)** Middle **d)** Proximal **e)** Arch

a)

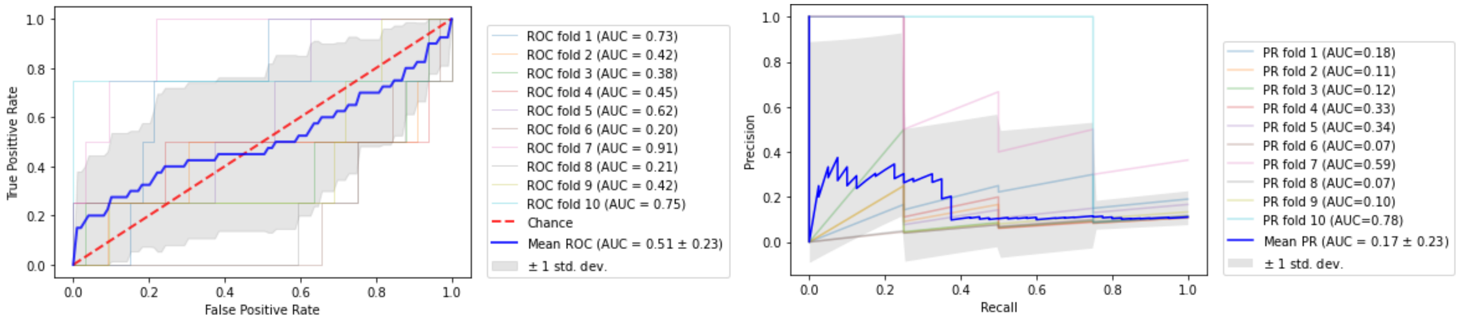

b)

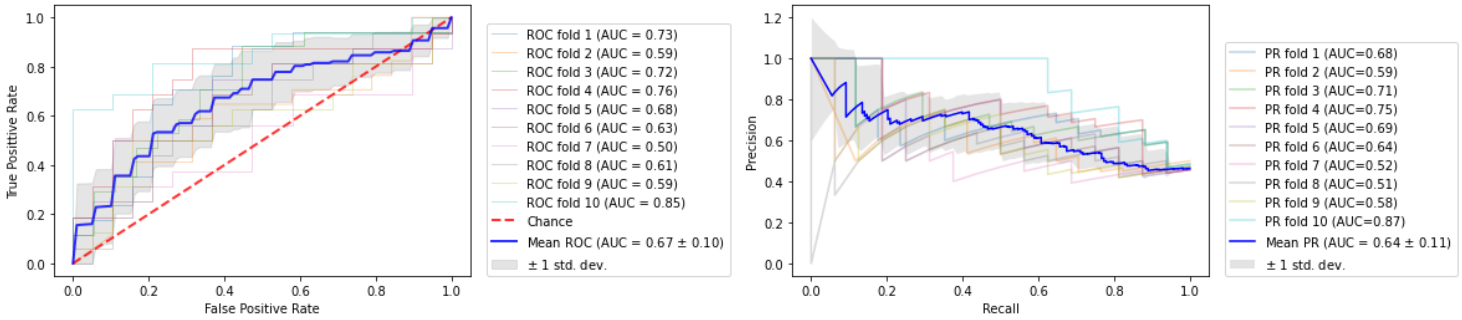

c)

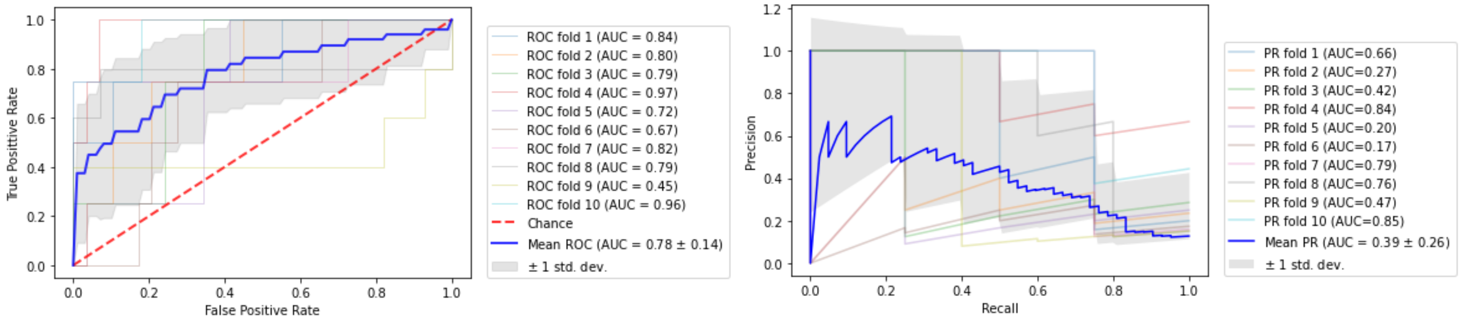

d)

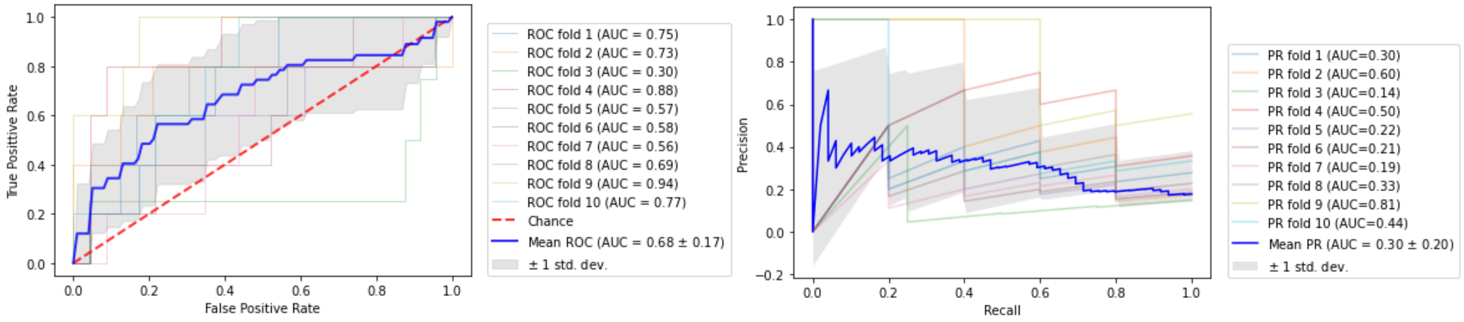

e)

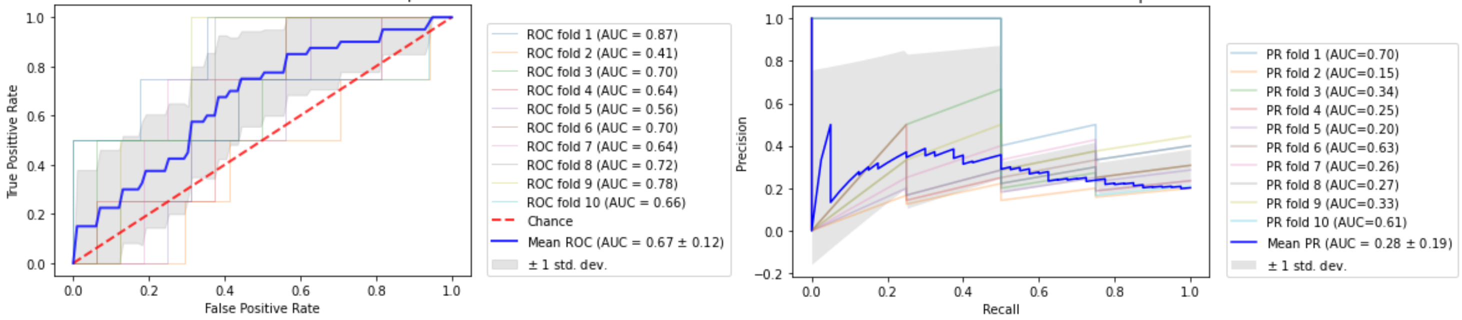

Supplementary Figure 7. The ROC (left) and PR curves (right) from 10-fold cross validation for the ViT trained on 374x128 spectrogram images for each location **a)** Anastomosis **b)** Distal **c)** Middle **d)** Proximal **e)** Arch

a)

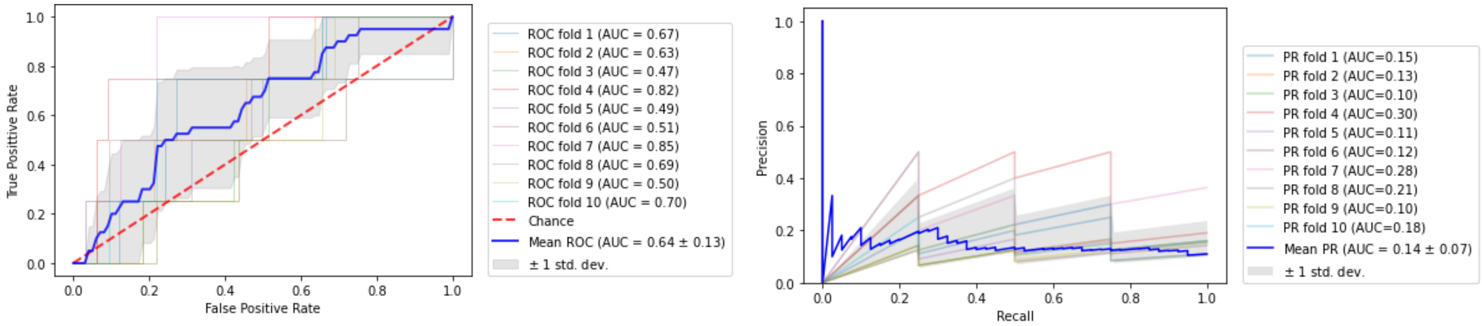

b)

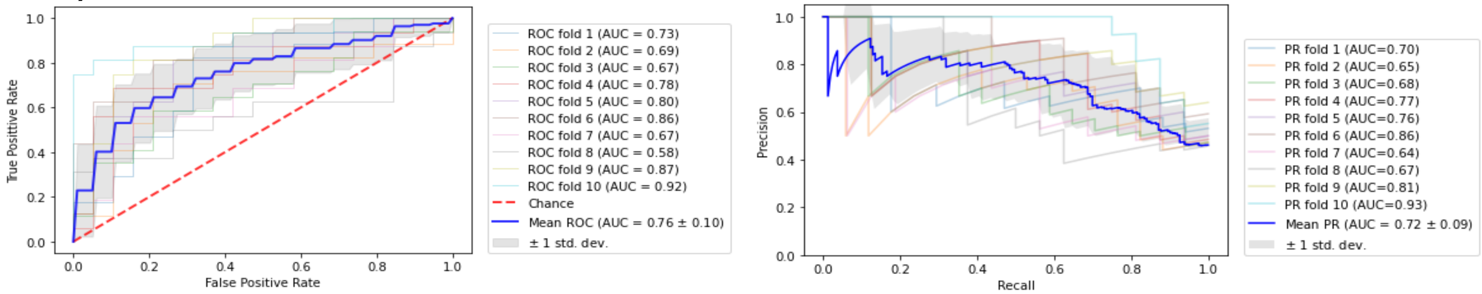

c)

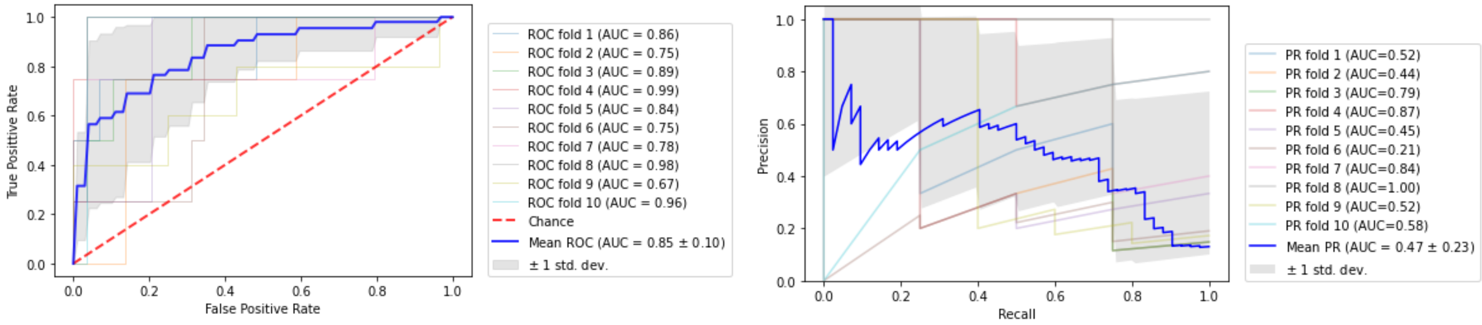

d)

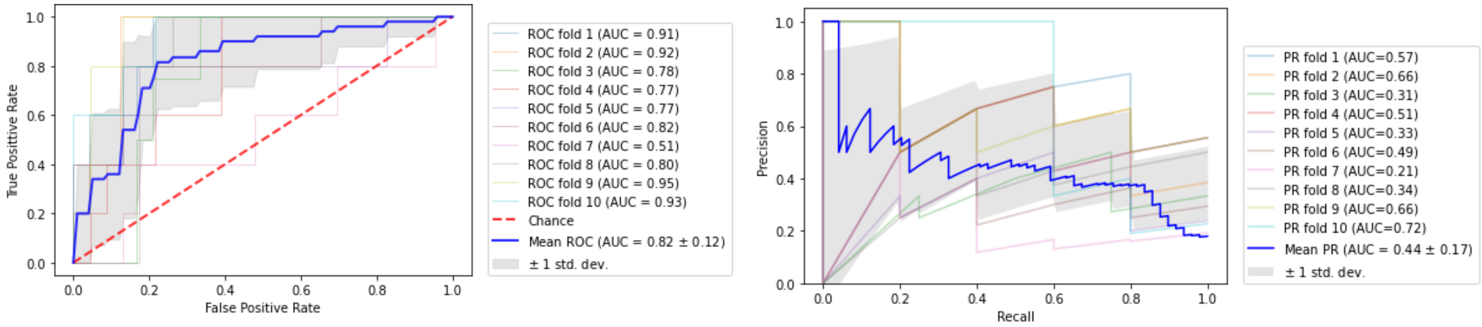

e)

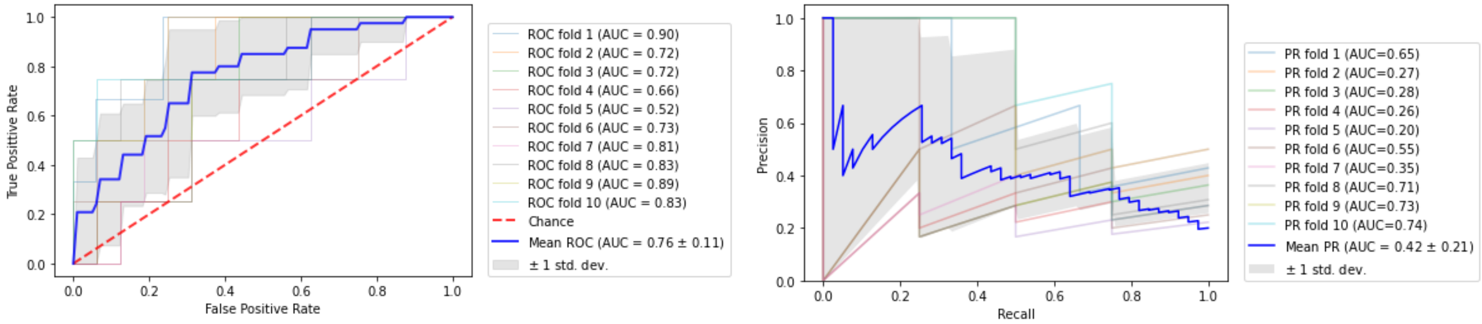

Supplementary Figure 8. The ROC (left) and PR curves (right) from 10-fold cross validation for the Vision Transformer trained on 128x128 recurrence plots for each location **a)** Anastomosis **b)** Distal **c)** Middle **d)** Proximal **e)** Arch

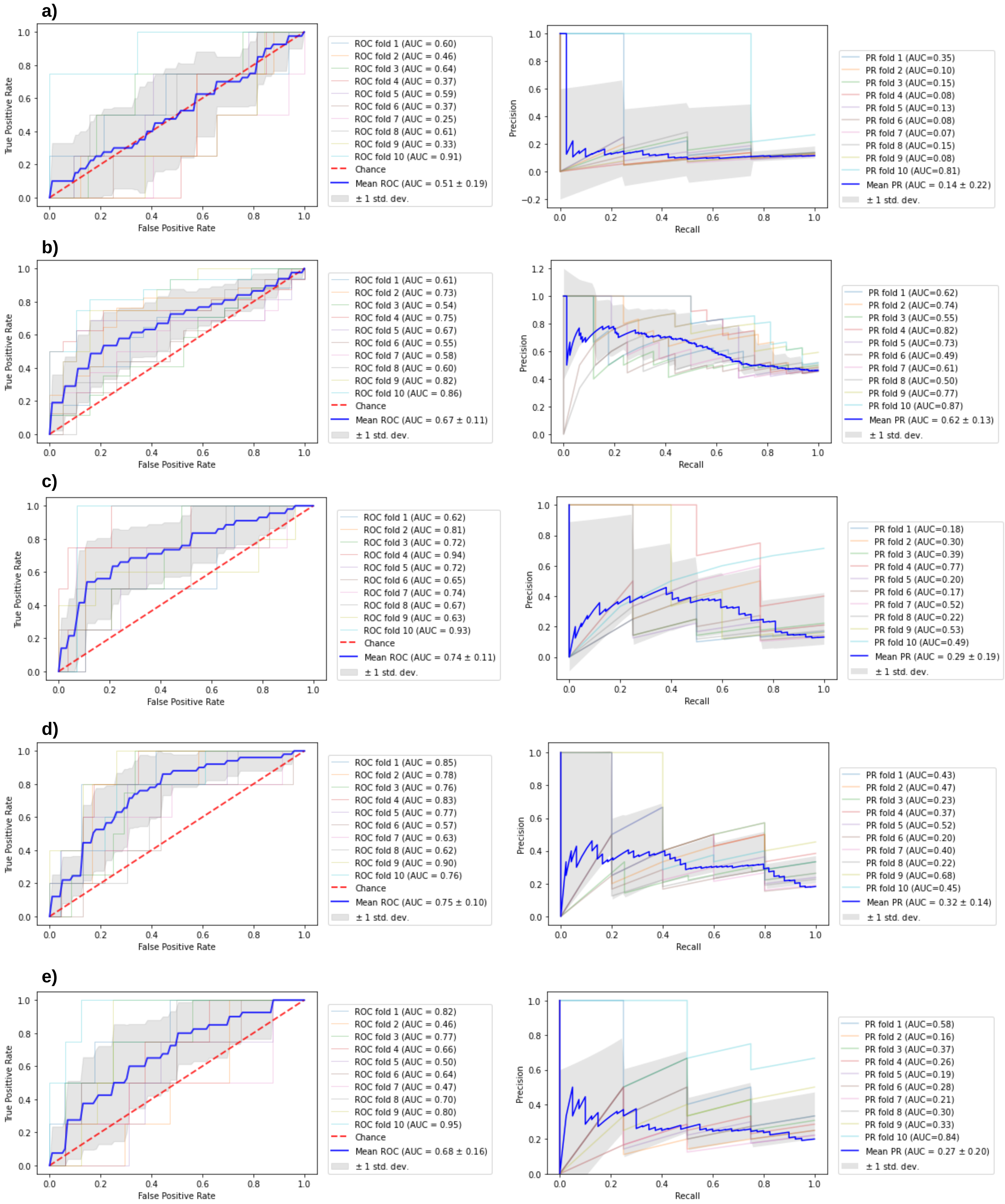

Supplementary Figure 9. ROC (left) and PRC (right) for Experiment 2: universal binary classifier to distinguish patent vs stenotic, with no location metadata, trained on 368x128 spectrogram images

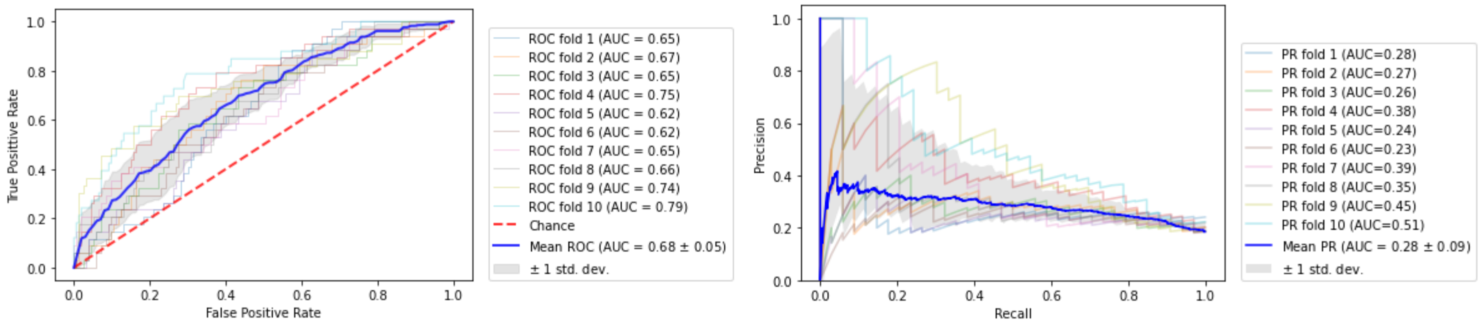

Supplementary Figure 10. ROC (left) and PRC (right) for Experiment 3: universal binary classifier to distinguish patent vs stenotic, with location metadata. The categorical location information is first one-hot encoded, then fed into an embedding layer that converts the one-hot encoded vectors into a dense numerical vector representation that is then concatenated to the flattened feature vector. The embedding layer is trained along with the ViT.

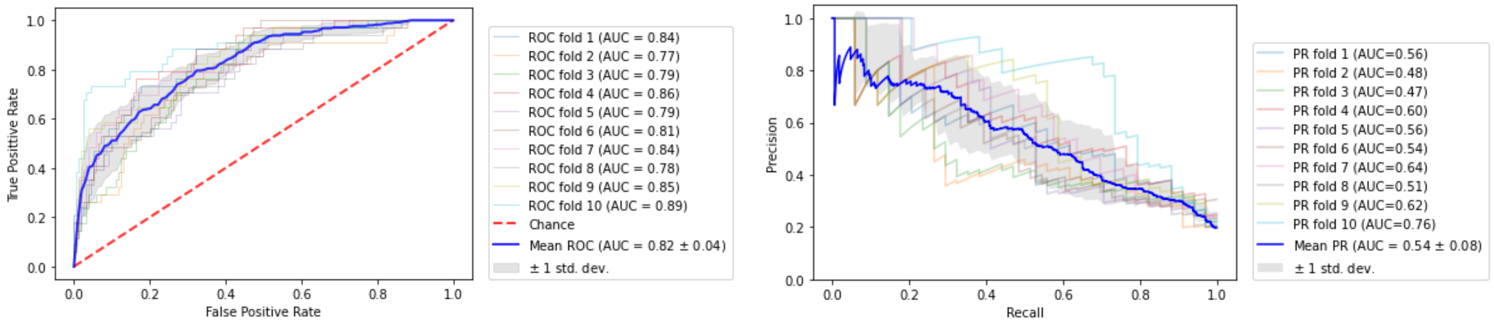

Supplementary Figure 11. Other categorical encoding methods for location metadata **a)** Modified ViT trained on 368x128 spectrogram images, with categorical location information encoded as integers: Artery:0; Arch: 1; Proximal: 2; Middle: 3; Distal 4: Anastomosis: 5. **b)** Modified ViT trained on 368x128 spectrogram images, with categorical location information encoded as integers: Artery:0; Arch: 10; Proximal: 20; Middle: 30; Distal 40: Anastomosis: 50. **c)** Modified ViT trained on 368x128 spectrogram images, with categorical location information encoded as integers: Artery:0; Arch: 100; Proximal: 200; Middle: 300; Distal 400: Anastomosis: 500. **d)** Modified ViT trained on 368x128 spectrogram images, encoding the categorical location information using one-hot encoding

a.

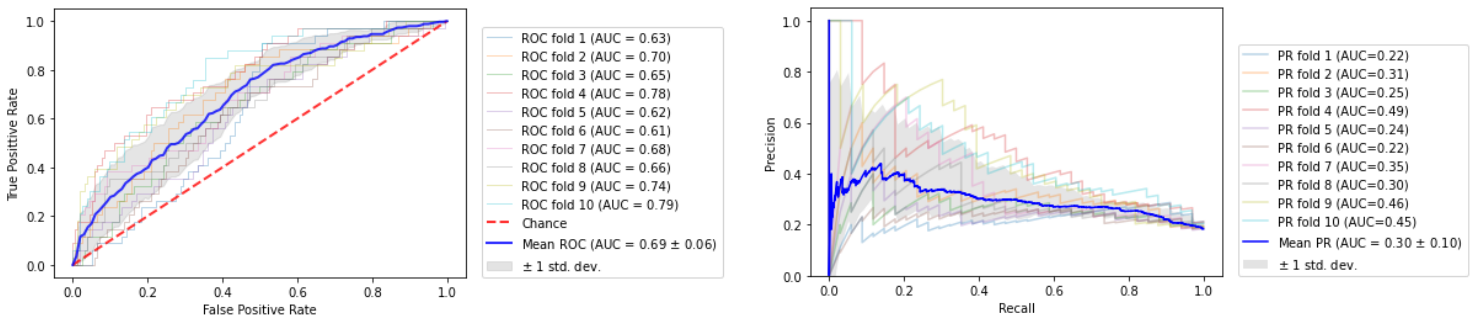

b.

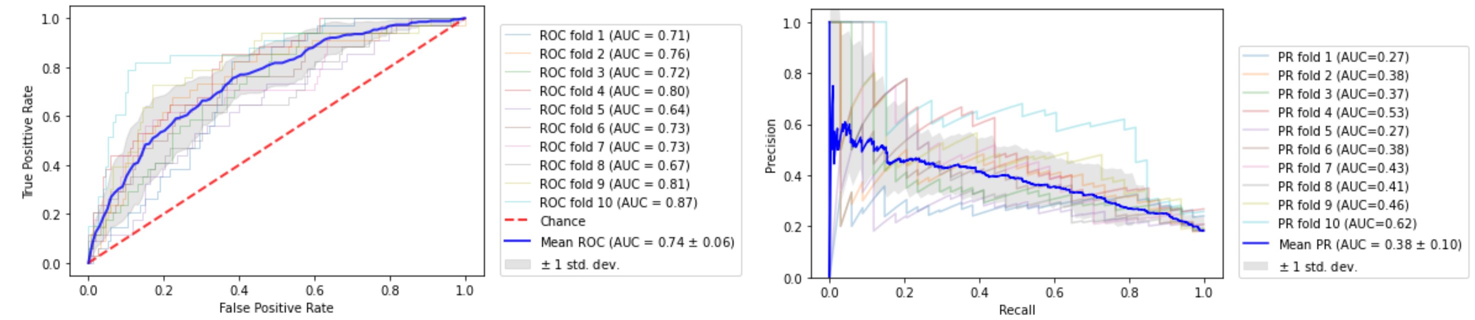

c.

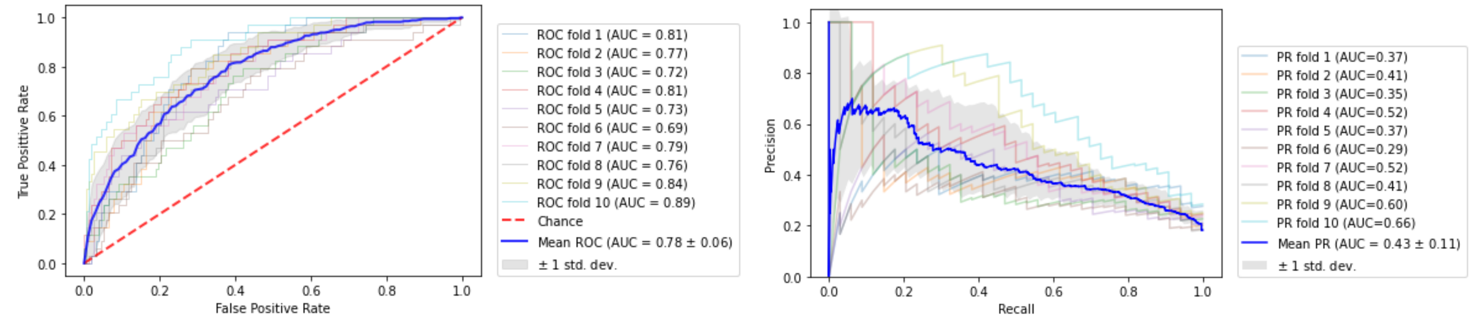

d.

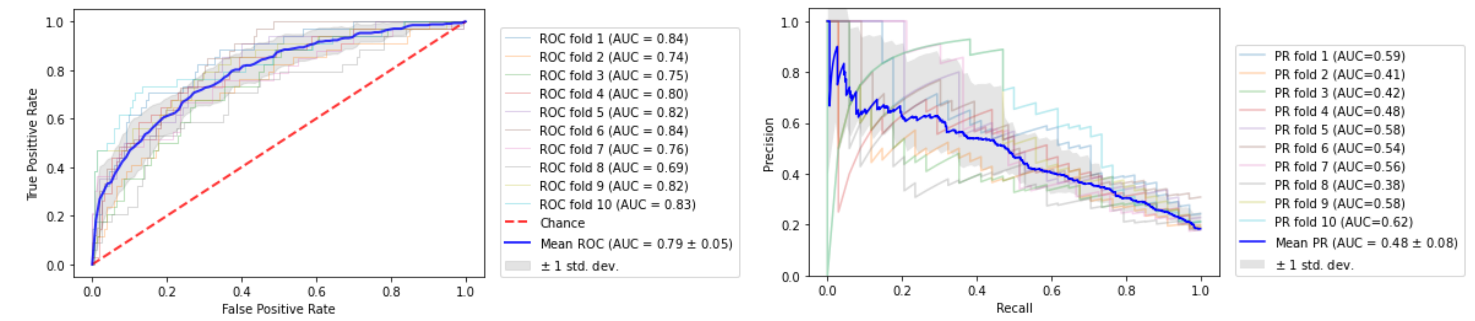

Supplementary Figure 12 Results from Experiment IV: Radial vs Brachial Artery

The resulting ROC curve from experiment IV, which seeks to train a ViT on 368x128 spectrogram images to distinguish radial vs brachial artery blood flow sounds at the “arterial” location is shown in Figure SI8 below.

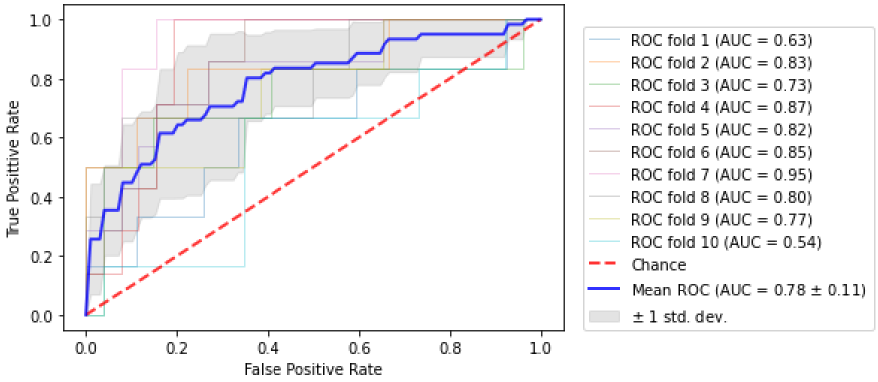

Supplementary Figure 13 Results from Experiment V: Cephalic vs Basilic vein

The resulting ROC curve from experiment V, which seeks to train a ViT on 368x128 spectrogram images to distinguish cephalic vs basilic vein blood flow sounds at the “venous arch” location is shown in Figure SI9 below.

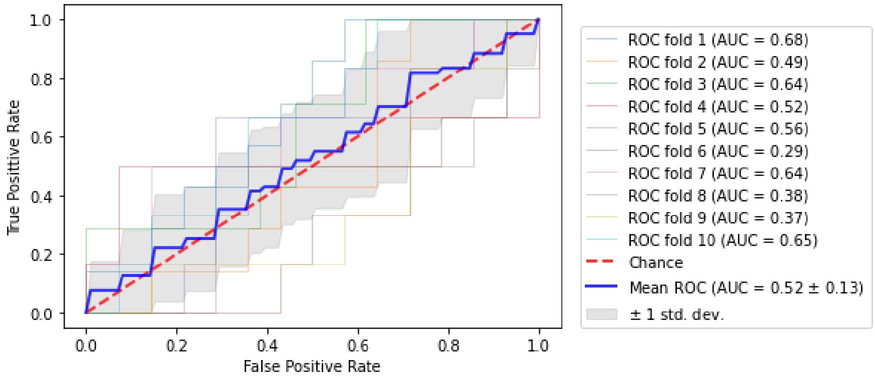

Supplementary Figure 14. The ROC (left) and PR curves (right) from 10-fold cross validation for the Vision Transformer trained on 368x128 spectrograms without using a weighted loss function for each location **a)** Anastomosis **b)** Distal **c)** Middle **d)** Proximal **e)** Arch

a)

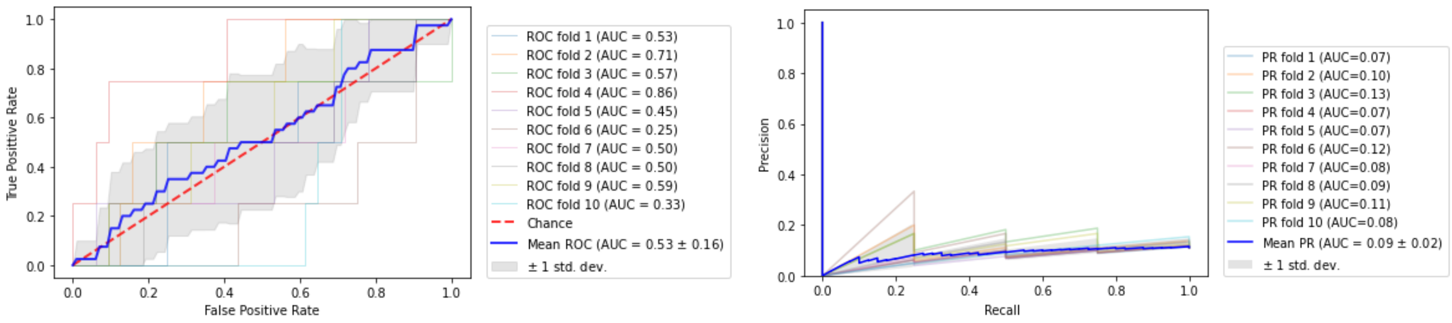

b)

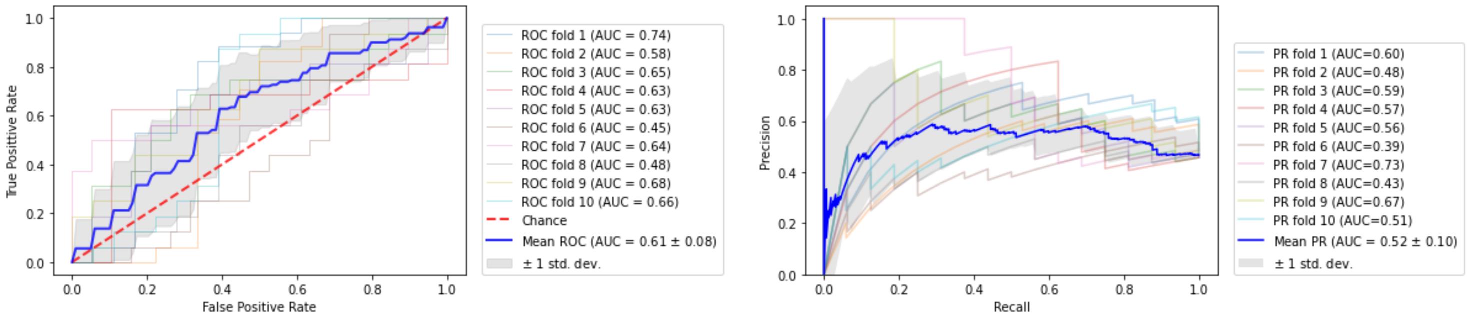

c)

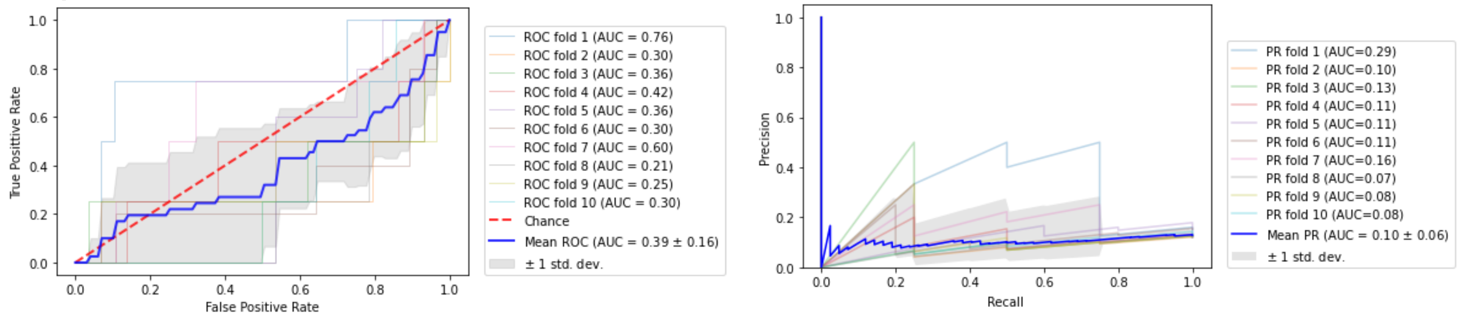

d)

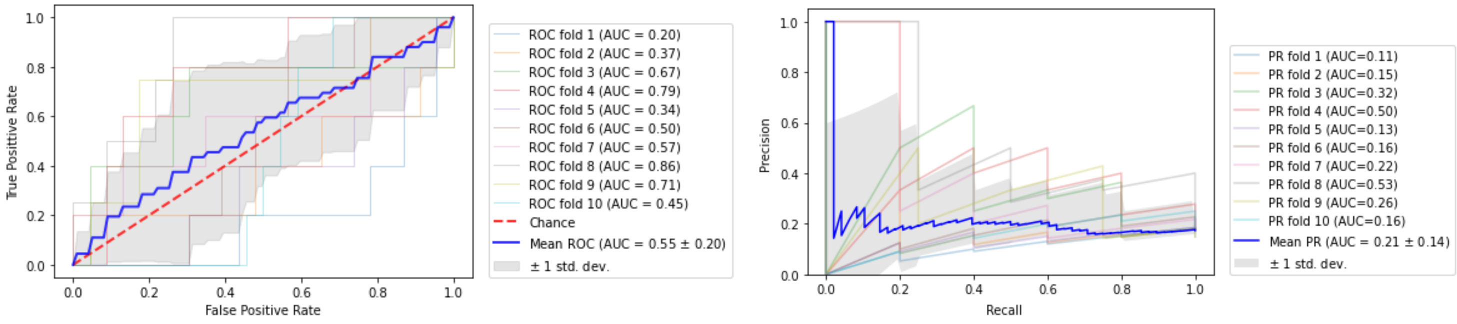

e)

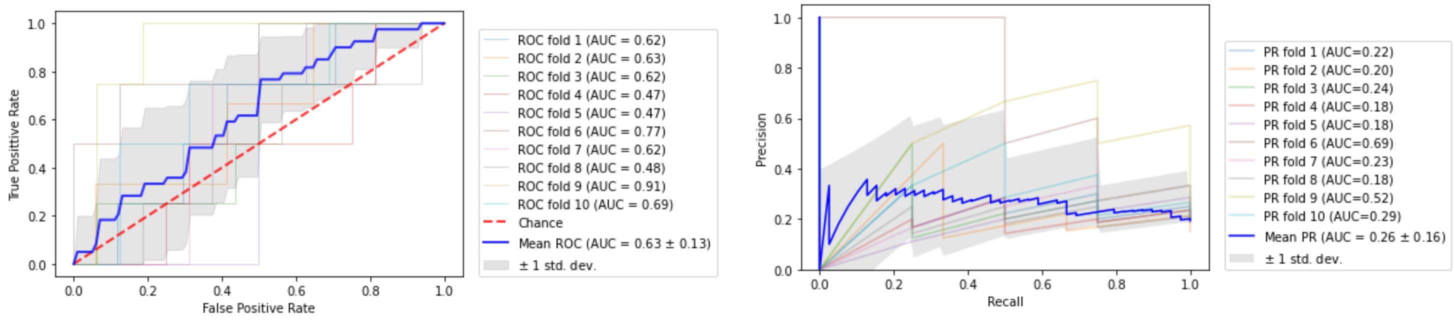

Supplementary Table 1. Results from Experiment VI: Independent Binary Classifiers to Distinguish Patent vs Stenotic at each Location, with Artery/Vein Anatomic Origin Metadata

The resulting AuROC and AuPRC values from 10-fold cross validation from experiment VI are shown in Table SI1. Here, the individual, location-based modified ViT is trained with categorical metadata encoding the anatomical origin of either the artery (1 for brachial artery, 0 for radial artery) or the anatomical origin of the vein (1 for cephalic vein, 0 for basilic vein).

**Artery Information**		
**Location**	AuROC	AuPRC
**Ana**	0.64±0.17	0.12±0.19
**Dist**	0.76±0.10	0.69±0.10
**Middle**	0.84±0.11	0.44±0.23
**Proximal**	0.81±0.13	0.41±0.18
**Arch**	0.72±0.15	0.33±0.24

**Vein Information**		
**Location**	AuROC	AuPRC
**Ana**	0.55±0.17	0.12±0.18
**Dist**	0.75±0.10	0.70±0.10
**Middle**	0.84±0.11	0.40±0.22
**Proximal**	0.83±0.11	0.44±0.20
**Arch**	0.73±0.15	0.34±0.20
